# ZnO nanostructured matrix as nexus catalysts for the removal of emerging pollutants

**DOI:** 10.1007/s11356-023-30713-3

**Published:** 2023-11-03

**Authors:** Ecaterina Matei, Anca Andreea Șăulean, Maria Râpă, Alexandra Constandache, Andra Mihaela Predescu, George Coman, Andrei Constantin Berbecaru, Cristian Predescu

**Affiliations:** 1 Faculty of Materials Science and Engineering, National University of Science and Technology POLITEHNICA Bucharest, 313 Splaiul Independentei, 060042 Bucharest, Romania; 2 Faculty of Biotechnical Systems Engineering, National University of Science and Technology POLITEHNICA Bucharest, 313 Splaiul Independentei, 060042 Bucharest, Romania

**Keywords:** Zinc oxide, Emerging pollutants, Nanocomposites, Green synthesis, Photocatalysts, Photodegradation, Water

## Abstract

Water pollution stands as a pressing global environmental concern, elevating the significance of innovative, dependable, and sustainable solutions. This study represents an extensive review of the use of photocatalytic zinc oxide nanoparticles (ZnO NPs) for the removal of emerging pollutants from water and wastewater. The study examines ZnO NPs’ different preparation methods, including physical, chemical, and green synthesis, and emphasizes on advantages, disadvantages, preparation factors, and investigation methods for the structural and morphological properties. ZnO NPs demonstrate remarkable properties as photocatalysts; however, their small dimensions pose an issue, leading to potential post-use environmental losses. A strategy to overcome this challenge is scaling up ZnO NP matrices for enhanced stability and efficiency. The paper introduces novel ZnO NP composites, by incorporating supports like carbon and clay that serve as photocatalysts in the removal of emerging pollutants from water and wastewater. In essence, this research underscores the urgency of finding innovative, efficient, and eco-friendly solutions for the removal of emerging pollutants from wastewater and highlights the high removal efficiencies obtained when using ZnO NPs obtained from green synthesis as a photocatalyst. Future research should be developed on the cost–benefit analysis regarding the preparation methods, treatment processes, and value-added product regeneration efficiency.

**Graphical Abstract:**

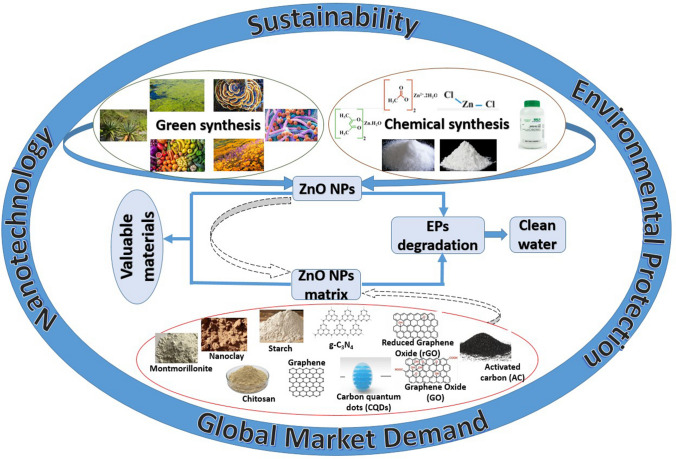

**Supplementary Information:**

The online version contains supplementary material available at 10.1007/s11356-023-30713-3.

## Introduction

The significance of water quality underscores the formulation of innovative design concepts for advanced nanomaterials, specifically tailored to address emerging pollutants in water bodies and wastewater treatment facilities (Al Ja’farawy et al. 2022). The aim is the creation of some facile and easily accessible products for rapid response and high sensitivity in pollution removal. The literature review indicates that industrial facilities annually release 300–400 million tons of waste into the world’s water, including organics and inorganics (Fanyun Chen et al. 2020a; Morasae Samadi et al. 2016). Nevertheless, pathogenic bacterial contaminants are also a major global health concern (M. Arunpandian et al. 2019). Due to the hazardous effects that solid and liquid wastes have on humans, animals, and aquatic environments, their disposal is essential (Munawar et al. 2020; Chongyang Liu et al. 2019; Jaspal Singh and Soni 2020).

The concerns related to the pollutants have been addressed by adapting various wastewater treatments, including physical adsorption, biological, and chemical techniques. These methods are difficult and ineffective at removing organic waste or harmful metal ions (Fanyun Chen et al. 2020a; Morasae Samadi et al. 2016). Current research has focused on advanced oxidation process (AOP) technologies that promise effective water purification (M. Samadi et al. 2019). Due to the effectiveness, low cost, and environmentally friendly approach, the use of photocatalysts is one of the AOP methods frequently employed for water treatment (M. A. Ahmed et al. 2019; M Arunpandian et al. 2020). When it comes to innovative physicochemical methods for the photodegradation of organic contaminants, semiconductor photocatalysts have attracted a lot of interest. They have a low energy requirement, are straightforward, and have mild reaction times (M. A. Ahmed et al. 2019; Pant et al. 2013).

Semiconductor metal oxides such as TiO_2_, ZnO, ZnO, ZnS, chalcogenides (CdS, CdSe), or Fe_2_O_3_ exhibit outstanding photocatalytic and antibacterial activity, making them the most promising materials in this field for the removal of organic pollutants and bacterial disinfection (Abebe et al. 2020). The use of nanoscale zero-valent iron (ZVI) in wastewater treatment has drawbacks, and difficulties like the separation of iron ox-hydroxide (FeOOH) nanoparticles in the treated water are caused by the release of soluble iron ions and susceptibility to surface oxidation, which is difficult and expensive to remove from wastewater (Simeonidis et al. 2016). Additionally, ZVI NPs have a tendency to form clusters when subjected to magnetic attraction and are capable of interacting with oxygen and compounds that include oxygen. ZVI NP toxicity to microbial species is another issue that raises questions because it may have an impact on both single cells and large ecosystems. Researchers have investigated a variety of approaches to overcome these difficulties, including strengthening ZVI nanoparticles with solid materials, changing ZVI nanoparticle physicochemical features, and modifying pH-related factors to accelerate the Fenton reaction (Aragaw et al. 2021).

TiO_2_ and ZnO, two semiconductor metal oxides with comparable band gaps (ZnO, 3.37 eV, and TiO_2_, 3.2 eV), make good photocatalysts for the purification of water (Pant et al. 2013; Raza et al. 2016). Due to its high excitation binding energy (60 meV), high electron mobility, high quantum efficiency, high photostability, non-toxicity, thermal stability, oxidation resistance, biosafety, and biocompatibility, ZnO is a possible replacement for TiO_2_ (Swati et al. 2020). Zinc (Zn) is a mineral nutrient that can have a positive impact on human health, whereas titanium (Ti) is a toxic heavy metal. ZnO is considered safe for contact with our skin and is even approved for use on baby skin. In contrast, TiO_2_ generates more free radicals that can potentially harm the skin. The key difference between these two oxides is that ZnO is a better UV absorber across more wavelengths when compared to TiO_2_. Numerous studies demonstrate that ZnO can exhibit efficiency comparable to TiO_2_ in the photocatalytic degradation of certain organic substances, and in some instances, ZnO exhibits even greater photocatalytic activity than TiO_2_ (G. G. Zheng et al. 2015). For example, Poulios et al. (Poulios et al. 2000) examined the photocatalytic degradation of basic yellow 2 dye, achieving a degradation rate of 95% after 60 min of exposure to UV light with TiO_2_ P-25 as the catalyst. Conversely, when ZnO was employed, the solution degraded almost completely, reaching nearly 100% degradation within the same 60-min period. The study by Muruganandham and Swaminathan (Muruganandham and Swaminathan 2006), which investigated the photocatalytic degradation of reactive yellow 14 dye in an aqueous solution, also showcased ZnO’s superior efficiency over TiO_2_. The research established the following reactivity order: ZnO > TiO_2_-P25 > TiO_2_ (anatase).

ZnO is an n-type semiconductor, which in the last decade has attracted attention due to the possibility of use in various fields such as optics and electronics, medicine, and the environment (Anbuvannan et al. 2015; Sundrarajan et al. 2015; Jamdagni et al. 2018). Compared to TiO_2_ or CuO, ZnO in the form of nanoparticles is a cheap, stable, and quickly obtained material (Jayaseelan et al. 2012). The semiconducting properties are given by the large band gap (3.37 eV) which offers superior catalytic, optical, anti-inflammatory, anticancer, antidiabetic, antibacterial, and antifungal properties, but also the possibility of filtering UV, being successfully applied in cosmetics (Mirzaei and Darroudi 2017; Patel et al. 2015).

Various applications derive from the morphology and structure of ZnO NPs prepared by various methods, so that nanoflakes, nanoflowers, nanobelts, nanorods, and nanowires are obtained (Paulkumar et al. 2014; Rajeshkumar et al. 2014). Research has demonstrated that the morphology of nanoparticles has a significant impact on their optical, electrochemical, sensory, thermal, and mechanical characteristics (Velasco et al. 2020). This influence on morphology is referred to as magnetic anisotropy (Fountain and Medd 2015). Additionally, the particle’s shape plays a role in the dispersion, degradation process, stability, and compatibility of ZnO nanoparticles (Garanin and Kachkachi 2003). The synthesis method used influences the morphologies the NPs obtained. Studies have shown that ZnO NPs prepared by the precipitation method can produce spherical and hexagonal morphologies (Velasco et al. 2020; Kolodziejczak-Radzimska and Jesionowski 2014). From hydrothermal synthesis, studies have reported bullet-like (100‒200 nm), rod-like (100‒200 nm), sheet (50‒200 nm), polyhedron (200‒400 nm), and crushed stone–like (50‒200 nm)-shaped ZnO NPs (Ismail et al. 2005; Dem’yanets et al. 2006).

The US Food and Drug Administration has also authorized ZnO as a safe antibacterial agent due to its efficient antibacterial activity, selectivity for bacterial cells, and low toxicity to human cells (Naskar et al. 2020). Many investigations were conducted to overcome some of the drawbacks of using ZnO for wastewater treatment, such as surface and structure modification, noble metal deposition, coupling carbon materials, formation of heterojunctions, and doping with metals and non-metals (Qi et al. 2017; Yadav et al. 2023; Folawewo and Bala 2022). ZnO is non-toxic, is environmentally friendly, and presents good stability properties; thus, it has been used for organic pollutant degradation (Phuruangrat et al. 2019; Tanji et al. 2023). Nevertheless, this approach falls short in enhancing photocatalytic activity, particularly in the visible light spectrum. Carbon materials serve as photoelectron reservoirs, storing and transporting the photogenerated electrons from ZnO to substrates, that is why they have undergone a tremendous increase in coupling to ZnO. The light absorption of ZnO expands into visible light and near infrared due to its photosensitizer characteristics (Qi et al. 2017).

Within these considerations, nanomaterials remain a promising solution especially when single particles are integrated into different selective and reliable matrix avoiding their loss in working environments. We present an extensive scientific investigation covering the period from 2000 to 2023 regarding ZnO nanostructures as reliable catalyst materials for the degradation of emerging pollutants from water sources. Although the literature presents numerous researches on the methods of obtaining ZnO, testing, and improvement solutions through ZnO integration in different matrix, the novelty of this research is to bring together those green methods that offer safety to the environment and aim to degrade emerging pollutants resulted from pharmaceuticals, personal care products, and other anthropic activities. In particular, we emphasized the integration concept of ZnO NPs into matrix as a low-cost tool for rapid degradation of emerging pollutants.

Our objectives for the present research were to (i) identify green syntheses as alternative for classical syntheses for ZnO, (ii) offer a screening analysis on EP types, (iii) analyze the degradation performances on EPs, and (iv) offer future perspectives.

## Methodology and results

For the present purpose, we extensively reviewed a large number of research papers as the most suitable methodological tool for conveying the correct data in the field to academic scholars. Thus, a comprehensive literature review ranging from theory to experimental results was analyzed. Besides these, our research included a number of existing guidelines referring to the literature reviews available today (Snyder 2019; Tranfield et al. 2003; Palmatier et al. 2018). Many of these guidelines describe different methodology approaches in order to achieve specific targets. Based on these considerations, the present literature research review was structured into 4 phases: design, conduct, analysis, and structuring and writing the review, as it is presented in Snyder’s paper (Snyder 2019).

**Fig. 1:**
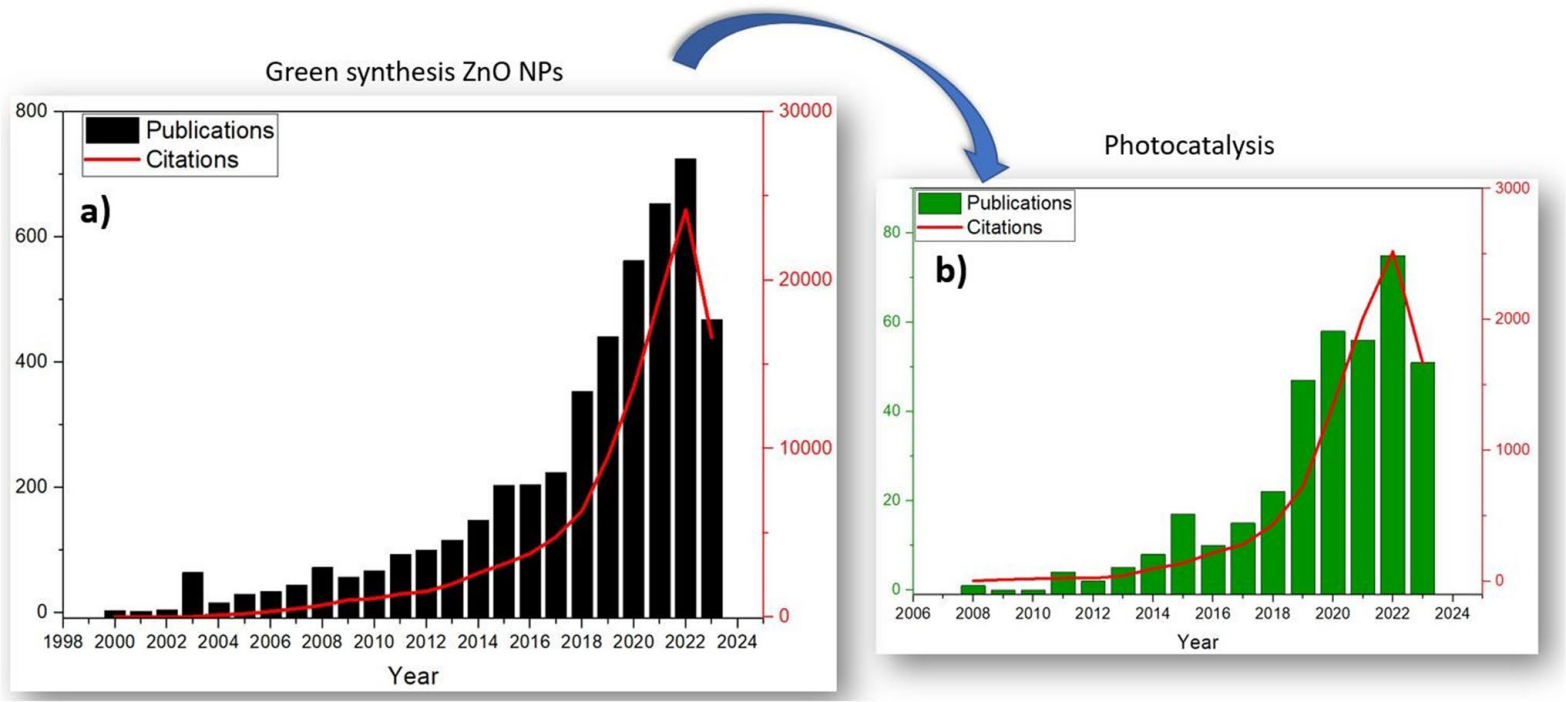
Scientific results generated between 2000 and 2023 regarding “ZnONPs Green synthesis” used in photocatalysis processes according to Web of Science database

The literature review was based on a number of publications between 2000 and 2023, according to the search engine of Web of Science. The primary keywords “green synthesis, ZnO” were chosen, for which a number of 4679 publications were recorded. According to the citations and publication number, it could be observed that in the last 10 years, there has been a continuous interest in the field of green synthesis for ZnO preparation. Thus, from only two or three papers at the beginning of 2000, a number of 725 papers were published in 2022. The number of citations was significant from 2020 where over 10,000 citations were recorded. Figure 1 includes these data from the Web of Science platform. By adding as the keyword “photocatalysis”, the number of papers was reduced to 371, with a constant tendency of the published articles since 2020 and a positive impact of the researches proved by the number of citations, as it can be seen in Fig. 1.

The selected publications, based on mentioned keywords, were collected and organized in accordance with the following criteria:

- Research design and methods of synthesis
- Photocatalysis performances for emerging pollutants
- Characterization investigation for ZnO NPs in order to validate structure and material performances

From the presented publications, an important impact for our research was identified at a number of 342 references that was chosen for EndNote as reference manager, with high impact as scientific information regarding ZnO NPs obtained with green synthesis and high efficiency as photocatalyst. The results of these publications are presented in the next chapters of this paper.

## Methods used for ZnO nanostructure preparation and their characterization

### Conventional syntheses

Based on conventional methods, ZnO NPs can be synthesized by applying controlled chemical and physical methods, such as sol–gel, sonochemical, solvothermal/hydrothermal, precipitation, microemulsion, and polyol (Verma et al. 2021). Physical methods involve physical processes such as colloidal dispersion, vapor condensation, amorphous crystallization, and physical fragmentation (Agarwal et al. 2017; Vidya et al. 2013; Aladpoosh and Montazer 2015; Chandrasekaran et al. 2016), using equipment to maintain adequate temperature and pressure. Chemical methods are recognized for the accuracy and reproducibility of the results, but they involve chemical agents that are often toxic and have a negative impact on the environment, so the interest in ecological methods is rapidly growing.

There are advantages and disadvantages for any of these methods, some being difficult to scale up, such as spray pyrolysis or low reproducibility for the sol–gel method or with a high consumption of solvents and reagents, as in the case of microemulsion or polyol methods.

#### Sol–gel synthesis method

The sol–gel method is a wet chemical method, being the most widespread method for the synthesis of photocatalytic semiconductors (Medina-Ramírez et al. 2015; Adeola et al. 2022; Thiagarajan et al. 2017). The final products can be crystalline and non-crystalline nanoparticles, ceramics, aerosols, and xerosols, depending on the final stage of thermal treatment. Precursor soil can be deposited on a substrate by coating or spin (Jamjoum et al. 2021). The method is cheap and allows control of the composition and the final product (Adeola et al. 2022). Through this method, semiconductors such as TiO_2_ can be doped with boron and nitrogen in order to develop materials with advanced properties applied to water decontamination processes with various pollutants, such as methyl orange (MO) (Gombac et al. 2007). Other examples would be obtaining hybrid nanocomposite magnesium aminoclay (MgAC)-Fe_2_O_3_/TiO_2_ used for the degradation of about 95% methylene blue (MB) and about 80% phenol from water (Bui et al. 2019).

Coupled oxide semiconductors of the p-n heterojunction-type ZnO/GO, Fe_2_O_3_/GO, ZnO/CuO, Nb_2_O_5_/TiO_2_, Ta_2_O_5_/TiO_2_, and SnO_2_/TiO_2_ were obtained by the sol–gel method, the metal oxide precursors being hydrolyzed under stirring, and the surface area of the metal oxide synthesized coupled leads to increased photocatalytic activity (Medina-Ramírez et al. 2015; Bayode et al. 2021a, b; Gajendiran and Rajendran 2014; Arbuj et al. 2013b, a; Nur et al. 2007).

The sol–gel technique is a versatile but complex method for preparing metal oxide nanoparticles. It involves a series of steps, including sol preparation, hydrolysis, polymerization, gel formation, solvent removal, and heat treatment, which can be time-consuming and intricate. One of the key challenges is achieving precise control over particle size and distribution, as uniformity and prevention of agglomeration can be difficult (Navas et al. 2021). Additionally, maintaining purity and minimizing contamination are crucial, as impurities from reagents or equipment can easily affect the quality of the final nanoparticles. The choice of precursor materials, cracking, shrinkage during drying, and the energy-intensive heating process further add to the complexities (Simon et al. 2009). Safety concerns and the need for specialized equipment and high-purity chemicals can also increase costs. Reproducibility can be a challenge due to the sensitivity of the process to various parameters (Modan and Schiopu 2020; Verma et al. 2021).

Despite these drawbacks, the sol–gel technique is valuable for tailoring metal oxide nanoparticles with unique properties. Researchers are actively working to refine the process and address these challenges to make it more efficient and reliable. Advancements in controlling particle size, minimizing impurities, and optimizing the process parameters are ongoing efforts to improve the utility of the sol–gel technique for nanoparticle synthesis.

#### Solvo-/hydrothermal synthesis method

The solvo-/hydrothermal method takes place in a closed reaction vessel called an autoclave, where high pressures can be obtained at relatively low temperatures, with the possibility to use different solvents like water, ethanol, or polyols (Yang and Park 2019; Islam et al. 2022). Apart from ensuring elevated product purity and crystalline quality, hydrothermal techniques regulate the ultimate nanostructure dimensions, configuration, and crystal phase within a minimally polluted closed system setting (Cavalu et al. 2018).

Through this method, control of morphology and crystallinity can be obtained for the fine powders. The solvothermal method is the simplest way to produce ZnO NPs, at low pressure and a temperature equal to or higher than the boiling point of the solvent used in the reaction. Depending on the polarities of the solvent, different morphologies can be obtained from nanorods, sheets, and even nanocomposites in which ZnO of about 5 nm is deposited in sheets of graphene (Kunjara Na Ayudhya et al. 2006; Lu et al. 2008; Wu et al. 2011).

The pH of the solution is important in the morphology of ZnO NP nanostructures, especially by the chemical precipitation method. Particle size decreases with increasing pH by dissociating OH ions at high pH (Magesh et al. 2018; Mahajan et al. 2019; Alias et al. 2010). The crystallinity and uniformity of the particles are also obtained by chemical precipitation, in an aqueous or non-aqueous medium, in the presence of a reducing agent, followed by calcination (ChangChun Chen et al. 2008; Ching-Fang Liu et al. 2018). As in the case of the hydro-/solvothermal method, the polarity of the solvent is important, and reproducible nanostructures can be obtained by adding a non-polar (hexane) or weakly polar (acetone) solvent which favors chemical precipitation of high surface area ZnO nanoparticles and reproducible morphological structures, but with a tendency to aggregate, which is why the stabilizing agent is also important (Halaciuga et al. 2011; Dutta et al. 2012). Low pH values lead to the dissociation of Zn^2+^ ions, in hydrothermal synthesis, the pH being almost neutral to alkaline to favor the hydrolysis of the Zn precursor in the presence of hydroxyl ions (Ching-Fang Liu et al. 2018). In the case of hydrothermal synthesis, the pH variation from 7 to 13 has an effect on the crystal growth rate and surface energy, obtaining various morphologies such as nanorods with hexagonal ends, spheroidal disc and hexagonal, porous hexagonal nanorods, and porous hexagonal nanorods assembled into nanoflower structures (Kumaresan et al. 2017).

The hydrothermal method uses high pressure and temperature, in the presence of which heterogeneous reactions take place in the presence of solvents (Adeola et al. 2022; Medina-Ramírez et al. 2015). TiO_2_ nanorods can be obtained (Muduli et al. 2011; Gao et al. 2015), CuO (Outokesh et al. 2011; Prathap et al. 2012), ZnO (Bin Liu and Zeng 2003; Gerbreders et al. 2020), MnO_2_ (Subramanian et al. 2005; Chu et al. 2017), etc. It is also possible to obtain hybrid composites used in degradation processes, such as TiO_2_ doped with boron and nitrogen for the degradation of bisphenol A (BPA) and TiO_2_-Bi_2_WO_6_ composite was developed for Rhodamine blue degradation (Abdelraheem et al. 2019; Hou et al. 2014).

**Fig. 2:**
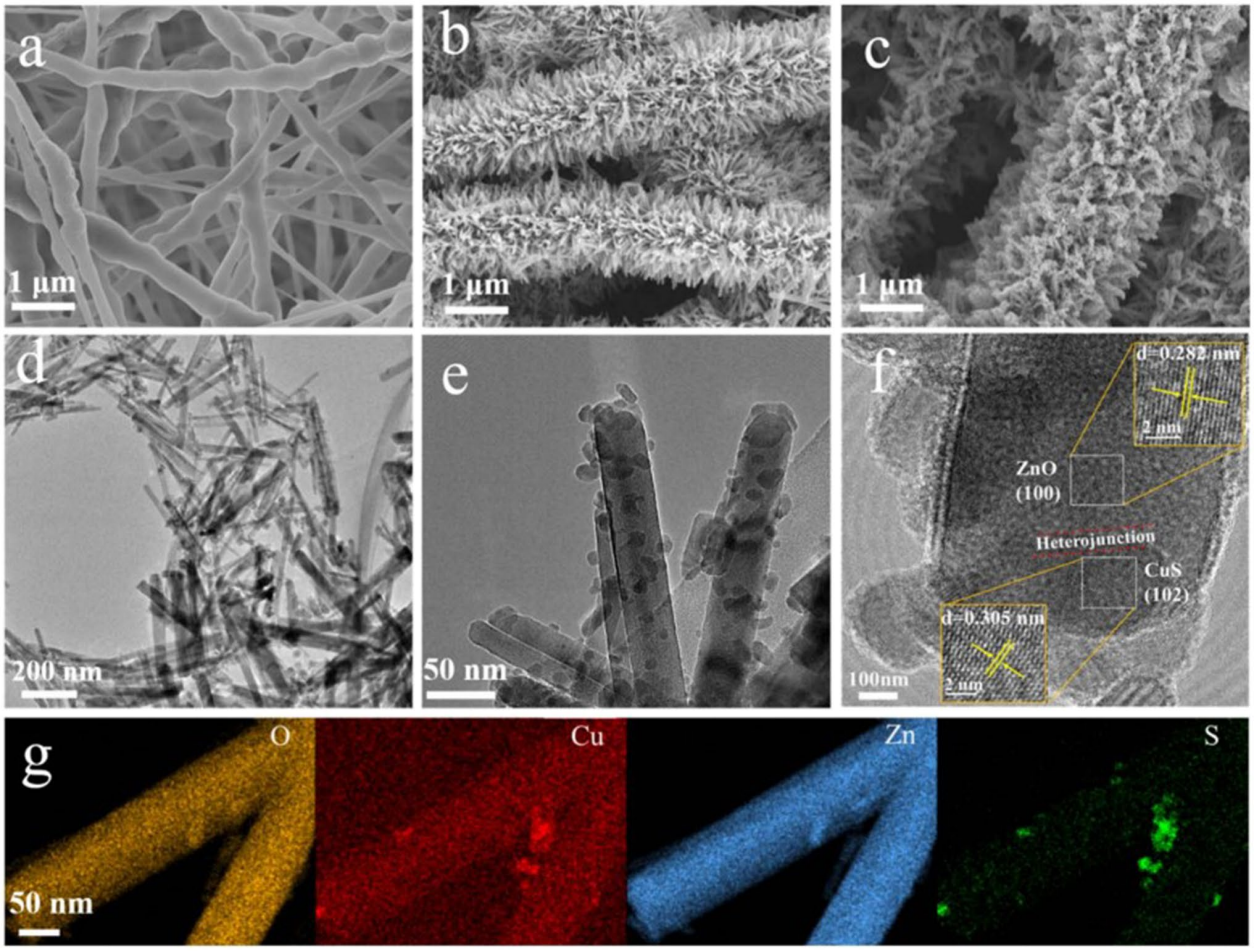
SEM images of **a** PVDF, **b** ZnO@PVDF, and **c** PVDF/ZnO/CuS nanocomposites. **d**–**f** TEM images and **g** EDX mapping of PVDF/ZnO/CuS nanocomposites (Zang et al. 2022) (open access)

Innovative photocatalysts composed of PVDF/ZnO/CuS were developed using electrospinning, hydrothermal treatment, and ion exchange techniques with the purpose to address the issue of particle aggregation in an aqueous environment (Zang et al. 2022). These photocatalysts demonstrated excellent stability during recycling and reuse. ZnO nanorods were firmly attached to PVDF nanofibers, serving as a support structure. Additionally, CuS NPs were introduced as photosensitizers to enhance the visible light photocatalytic efficiency and compensate for the relatively low quantum efficiency of ZnO. The results demonstrated superior photocatalytic performance in the degradation of MB under both UV and visible light, with kinetic constants of 9.01 × 10^−3^ min^−1^ for UV irradiation and 6.53 × 10^−3^ min^−1^ for visible light. Before the hydrothermal treatment, the morphology of PVDF nanofibers appeared relatively smooth, with each nanofiber having a diameter of approximately 300 nm (Fig. 2a). However, the diameter distribution was somewhat uneven. After the hydrothermal process, a multitude of neatly arranged ZnO nanowhiskers enveloped the nanofibers (Fig. 2b), significantly increasing their specific surface area. Subsequently, in situ reduction techniques uniformly distributed CuS nanoparticles on the ZnO nanorods (Fig. 2c). Transmission electron microscopy (TEM) images revealed crystal spacings of 0.282 nm and 0.305 nm, corresponding to the (100) crystal plane of ZnO (wurtzite-type) and the (102) crystal plane of CuS (Fig. 2d, e). The interface between ZnO and CuS, marked with a red line, confirmed the successful construction of the p-n heterojunctions (Fig. 2f).

As the process is considered environmentally advantageous, it is incorporated into eco-friendly approaches for synthesizing ZnO NPs. Nevertheless, this approach comes with certain drawbacks. For instance, it necessitates the use of a highly costly autoclave and imposes restrictions on research due to the inability to keep the reactor open. Other disadvantages are represented by the toxicity of some solvents that are used in this process; the reactions can take place in long periods of time (5–48 h) (Verma et al. 2021).

#### Co-precipitation synthesis method

The co-precipitation method generates metallic NPs through concurrent nucleation, subsequent growth, and eventual agglomeration of very small nuclei. The solution’s pH is modified within a designated range, usually falling between 7 and 11, to trigger the precipitation of zinc ions (Naser et al. 2023). Subsequently, the resulting blend is either stirred or subjected to sonication for a specified duration to encourage the development of ZnO NPs. One of the merits associated with the co-precipitation technique is its straightforwardness and cost-effectiveness. This technique offers various advantages, such as simplicity of application, reduced reliance on high temperatures, and straightforward energy control (Rane et al. 2018). Additionally, this method can yield a substantial quantity of ZnO nanoparticles with high efficiency. However, it is important to note that this approach has a notable disadvantage: it results in nanoparticles with a significant presence of attached water molecules. Furthermore, it exhibits drawbacks such as batch-to-batch repeatability challenges, a broad spectrum of particle sizes, and pronounced agglomeration tendencies (Mostafavi et al. 2015). The necessary materials are easily obtainable, and the synthesis procedure is comparatively uncomplicated (Marciello et al. 2016).

The precipitation reagent influences the nucleation speed, morphology, and crystallinity of the formed ZnO particles. Crystallinity decreases with increasing rate of precipitating agent volume (e.g., NaOH), and decreasing precipitating agent volume per minute leads to the change of morphology from ZnO NPs to nanorods (Bekkari et al. 2017). Different co-precipitation methods were employed to synthesize ZnO NPs with different particle sizes. One method utilized zinc acetate solution in methanol, resulting in spherical ZnO NPs ranging from 2 to 10 nm in size. Another approach involved zinc acetate dihydrate, hydrochloric acid, and ammonia as reactants, yielding pseudo-spherical ZnO NPs with an average size of 11 to 20 nm (Purwaningsih et al. 2016). Additionally, a similar co-precipitation technique was employed by Adam et al. to produce ZnO NPs with an average diameter of 140 nm (Adam et al. 2018).

High degradation efficiencies under visible radiation were obtained for the ZnO:Au photocatalyst when it was used for the degradation of chloroquine phosphate (CLQ), paracetamol (PAR), diclofenac sodium (DCF), and ciprofloxacin (CIP) pharmaceuticals in water (F. Y. Zheng et al. 2023) and W/Ag/ZnO nanocomposite created for degradation of Turquoise Blue Dye (TBD) (Noreen et al. 2022).

#### Sonochemical synthesis method

The sonochemical method is based on a physical phenomenon of acoustic cavitation, through which nanometals, oxides, semiconductors, metal alloys, etc. are obtained (Medina-Ramírez et al. 2015; Hangxun Xu et al. 2013). The method is advantageous economically and for the environment, and the shape and size of the materials can be controlled (Abbas et al. 2014; Ali Dheyab et al. 2021). A major drawback of this method is that it has low efficiencies (Modan and Schiopu 2020).

The literature indicates the obtaining of CdO nanorods which in the presence of Ag can lead to the formation of Cd(OH)_2_ with Ag nanodots deposited on the surface (Abbas et al. 2014). ZnO NPs were also obtained by the sonochemical method with zinc acetate precursor and a solvent that acts as the base and stabilizer and template for ZnO NPs (Bhatte et al. 2012; Nandi and Das 2020). The literature also indicates that ultrasonic waves play a crucial role in facilitating the conversion of Zn(OH)_2_ into single-phase ZnO NPs.

Through sonochemical synthesis, the crystallinity and size of ZnO NPs depend on the ultrasonic wave intensity, sonication time, and solvent type (Hangxun Xu et al. 2013; Banerjee et al. 2012; Alammar and Mudring 2011; Zak et al. 2013). In this way, 0-D, 1-D, 2-D ZnO nanostructures, nanoflowers, and 3-D nanoflakes can be obtained (Ghosh et al. 2014; Verma et al. 2021). The pH variation with the sonochemical method led to spherical shapes at pH 9.5, and by increasing to about 11, the shapes became ellipsoidal and respectively rod or sheet at pH 12.5 (Xiao et al. 2008).

**Fig. 3:**
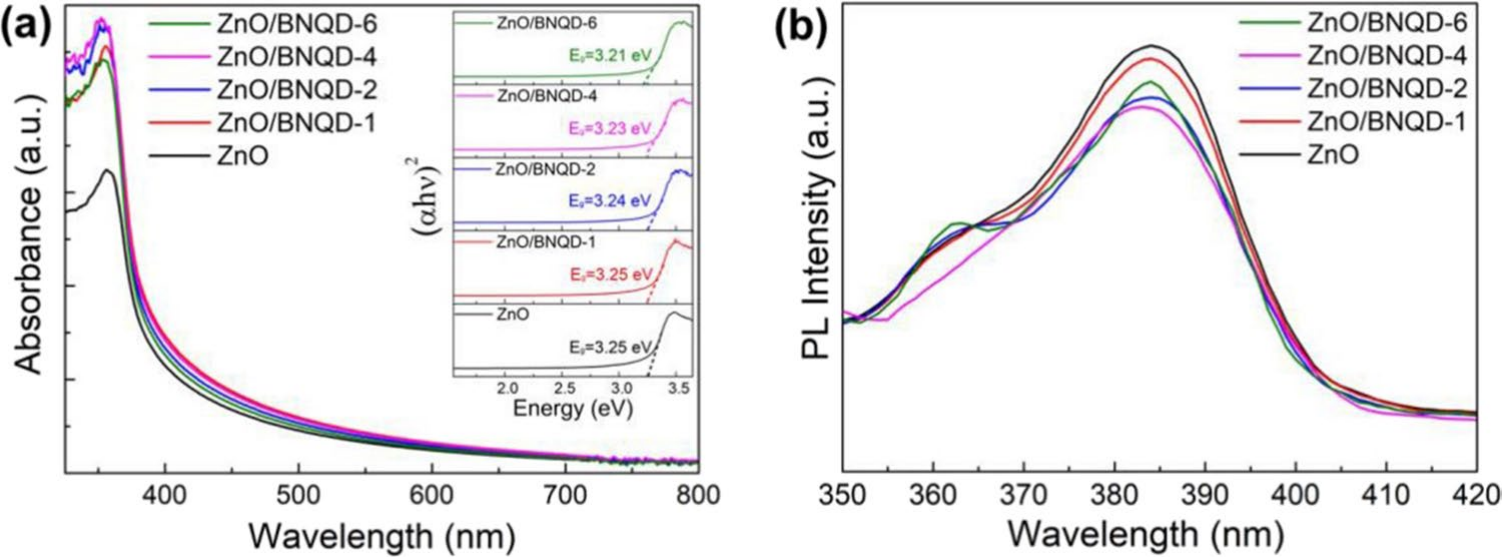
Absorbance (**a**) and PL (**b**) spectra for ZnO/BNQD_*x*_ composites (D. Liu et al. 2022) (open access)

A successful synthesis was achieved through a sonication process, resulting in a heterostructure photocatalyst comprising ZnO NPs decorated with boron nitride quantum dots (ZnO/BNQD_*x*_) (D. Liu et al. 2022). The ZnO/BNQD_*x*_ (*x* = 1, 2, 4, and 6 wt.%) nanocomposites showed an enhanced photocatalytic activity in the degradation of methylene blue (MB) and methyl orange (MO), attributed to the formation of a heterojunction, which facilitates effective hole extraction by BNQD_*x*_ while simultaneously preventing the recombination of photoinduced charge carriers. The optical characteristics of both ZnO and ZnO/BNQD_*x*_ nanocomposites were explored using UV–visible diffuse reflectance spectroscopy and photoluminescence (PL) analysis, as displayed in Fig. 3a and b. ZnO exhibits distinct UV absorption extending up to around 360 nm, corresponding to a 3.25 eV band gap. The ZnO/BNQD samples exhibit a visible range absorption with increasing absorption intensity as the BNQD quantity rises. Consequently, the band gap decreased gradually to 3.25 eV (ZnO/BNQD-1), 3.24 eV (ZnO/BNQD-2), 3.23 eV (ZnO/BNQD-4), and 3.21 eV (ZnO/BNQD-6), indicating that the interaction between ZnO and BNQDs enhanced visible light absorption, thus improving the photocatalyst’s activity in visible light (Fig. 3a). The PL analysis suggests improved separation of photoinduced electron–hole pairs due to the formation of a heterojunction between ZnO and BNQD_*x*_ (Fig. 3b).

#### Microemulsion synthesis method

In a microemulsion environment, water droplets collided, triggering a precipitation reaction that resulted in the formation of NPs stabilized by surfactants (Islam et al. 2022). This approach is favored for its simplicity, thermodynamic stability, and low residue. However, microemulsion techniques have drawbacks such as sensitivity to temperature and pH, as well as the continuous need for high surfactant concentrations, which can be irritating (Rane et al. 2018). Wang et al. produced ZnO NPs in a microchannel reactor with an average diameter of 16 nm, followed by drying and calcination (Y. Wang et al. 2014). Similarly, Li et al. generated ZnO NPs through a straightforward microemulsion process, yielding NPs with various shapes, including columnar and spherical morphologies (X. C. Li et al. 2009).

**Fig. 4:**
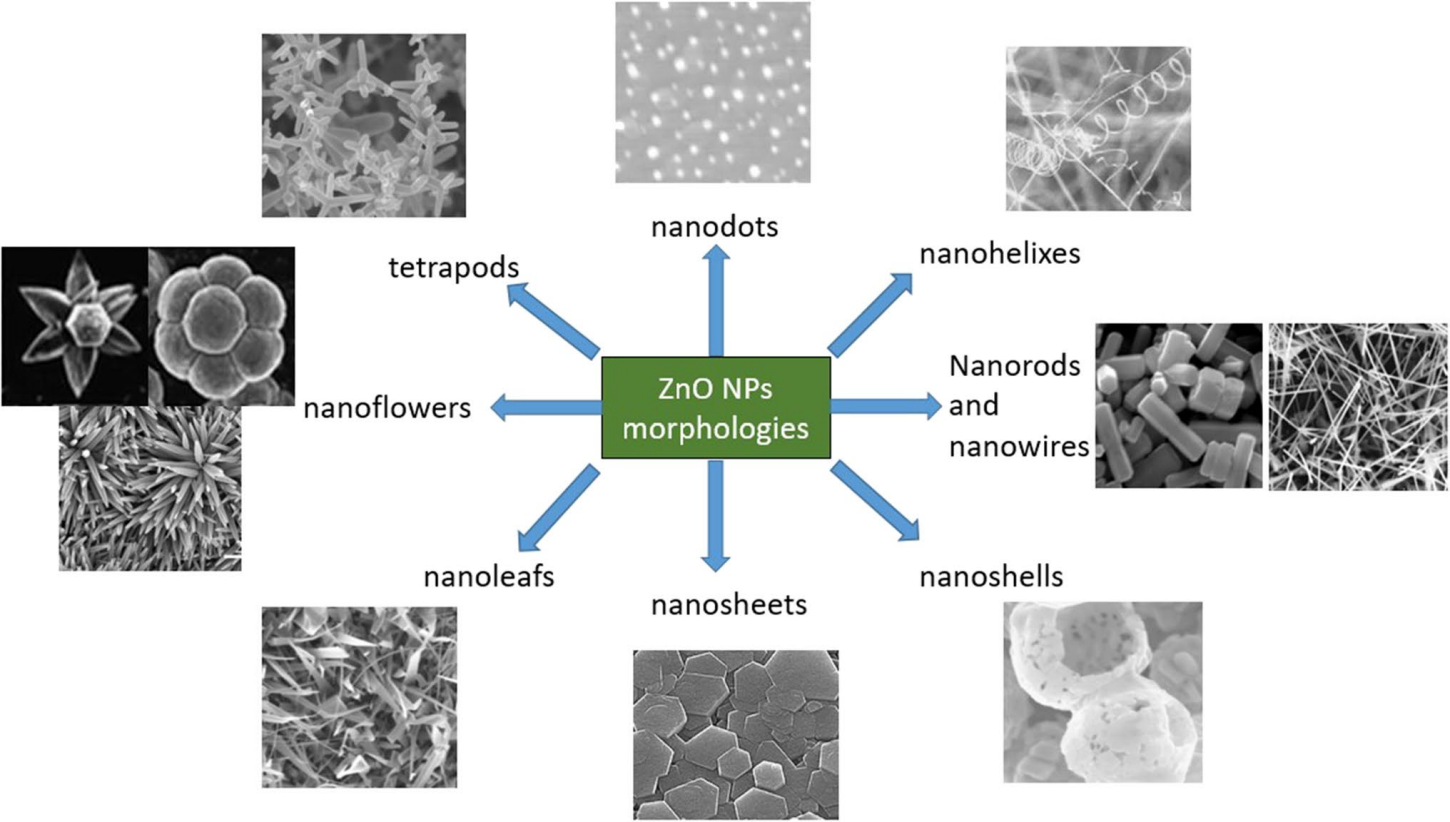
Different morphologies of ZnO NPs

The synthesis of ZnO NPs by microemulsion offers a control of purity and crystallinity by using organic solvents immiscible with the aqueous solution of the metal solution, in the presence of anionic and cationic surfactants (Verma et al. 2021; Sarkar et al. 2011; Atul B Lavand and Malghe 2015a). Nanostructures such as spheres, needles, and rods can be obtained, as it can be observed in Fig. 4. The use of organic solvents possessing multiple hydroxyl groups was recently implemented through the polyol method, through which nanorods can be obtained under controllable conditions, using zinc acetate precursor in diethylene glycol at temperature, in the presence of capping agents such as polyvinylpyrrolidone or p-toluene sulfonic acid (Alves et al. 2018; Anžlovar et al. 2012; Flores-Carrasco et al. 2019; Lee et al. 2008; Mei Wang et al. 2018).

#### Microwave-assisted synthesis method

The microwave-assisted method is a popular choice for synthesizing nanomaterials due to its numerous advantages compared to other traditional methods and is commonly used for producing ZnO NPs (Wojnarowicz et al. 2020). In this technique, microwave radiation is employed to trigger the reaction between zinc acetate or zinc nitrate and a base like sodium hydroxide or ammonium hydroxide in a suitable solvent such as water or ethanol (Kolodziejczak-Radzimska and Jesionowski 2014; Prommalikit et al. 2019). The process is conducted under controlled microwave conditions, including temperature and power. This method stands out for its rapid and efficient production of ZnO nanoparticles, with the ability to complete the synthesis within minutes to hours. Moreover, it offers the advantage of tailoring nanoparticle size and morphology by adjusting reaction parameters.

The rapid synthesis of thin films of ZnO NPs, advantageous to avoid the risk of uncontrolled release of NPs, can be achieved by pyrolysis by spraying the metal precursor in aerosol form on a hot solid substrate, using a carrier gas at high pressure (D Sumanth Kumar et al. 2018a). Homogeneous spheres of ZnO NPs of about 20–30 nm can be obtained (Turner et al. 2010; Tani et al. 2002). Microwave radiation ensures fast and uniform heating of the solution compared to classical heat treatment (Bilecka and Niederberger 2010). The irradiation power of the furnace on the solution of metal precursors ensures different morphologies of ZnO NPs obtained from nanoflower shapes to 1D nanoneedles, and the higher the reaction speed, the faster the growth of ZnO nuclei occurs (Barreto et al. 2013).

The rapid microwave synthesis method proved effective in producing zinc oxide nanorods (ZnO NRs) capable of absorbing visible light photons (Cardoza-Contreras et al. 2019). Introducing silver (Ag) and gallium (Ga) into the ZnO nanorods had distinct impacts on their optical properties. At low concentrations, Ga enhanced the defect band of ZnO NRs, whereas higher concentrations increased the intensity of the near band edge (NBE) emission. In the experiment conducted to degrade methylene blue (MB), it was observed that a 0.1% Ga doping significantly improved the photocatalytic performance of ZnO NRs. This improvement in photocatalytic efficiency suggests that the low-level Ga doping creates more surface defects, which effectively trap photogenerated electrons and holes, reducing their recombination. On the other hand, low-level silver doping increased the intensity of both the NBE emission and defect band, possibly indicating an increase in lattice defects. These defects can act as recombination centers, resulting in a slight reduction in photocatalytic activity.

ZnO nanostructures were integrated with reduced graphene oxide (ZnO-rGO) through a one-pot microwave-assisted hydrothermal synthesis as a promising approach for polychlorinated biphenyl (PCB) degradation (Merlano et al. 2022). As a result, the composites displayed enhanced photocatalytic efficiency for PCB degradation in contrast to ZnO NPs. Achieving complete PCB mineralization is seldom documented, necessitating prolonged irradiation durations. High removal rates (> 90%) and under scalable experimental conditions were reported.

**Fig. 5:**
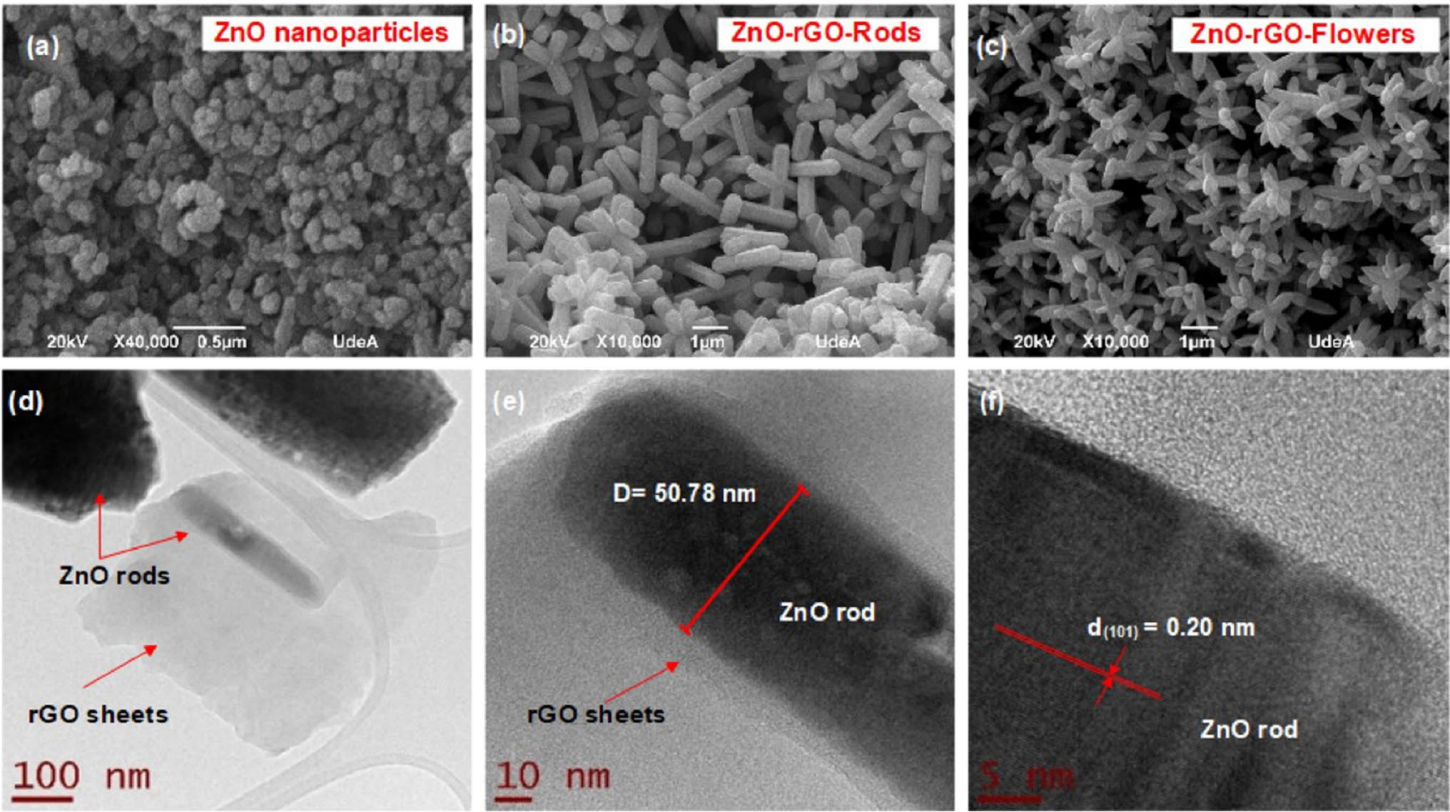
SEM micrographs of **a** ZnO nanoparticles, **b** ZnO-rGO-rods, and **c** ZnO-rGO-flowers. **d**–**f** TEM micrographs of ZnO-rGO-rods (Merlano et al. 2022) (open access)

The SEM and TEM analyses (Fig. 5) were conducted to examine the morphologies of the synthesized materials. ZnO NPs were successfully generated, exhibiting an approximate spherical shape with an average particle size of 108 nm (Fig. 5a). In the case of the nanocomposites, distinct ZnO nanostructures in the form of rods and flowers were achieved, completely covering the rGO sheets (Fig. 5b, c). The rod-shaped ZnO nanoparticles that adhered to the rGO flakes had an average length of 2.60 µm and an average diameter of 511 nm. TEM micrographs clearly show the anchoring of a ZnO rod within the rGO sheets (Fig. 5d, e). Figure 5f provides evidence of an estimated d-spacing value of 0.20 nm between two adjacent lattice planes, corresponding to the (101) planes of hexagonal wurtzite ZnO which can be observed.

#### High-energy ball milling synthesis method

The high-energy ball milling (HEBM) synthesis method for ZnO nanoparticles offers advantages such as controlled size and enhanced properties but is accompanied by notable drawbacks (Hodaei et al. 2015). It involves specialized, potentially costly equipment and high energy consumption, and the use of milling balls can introduce contamination (Piras et al. 2019). The process can be time-consuming, result in a broad size distribution, generate heat, cause mechanical damage, and pose challenges for scale-up. However, researchers still value HEBM for its ability to tailor nanoparticle properties, especially when precise control is crucial for specific applications, weighing its advantages against these disadvantages based on project requirements.

Researchers have utilized this technique to synthesize ZnO NPs using commercially available ZnO powder with an initial mean particle size of 0.8 nm (Prommalikit et al. 2019). Varying milling durations resulted in ZnO NPs with final sizes ranging from 20 to 400 nm, with longer milling times leading to smaller particle sizes. For instance, spherical ZnO NPs with an approximate size of 30 nm were obtained after a specific milling duration.

Photocatalysis is a green process of advanced oxidation that takes place on the basis of light that decomposes organic contaminants in the presence of nanostructured oxide materials and immobilizes microbial agents in water (Bora et al. 2017).

#### Laser ablation synthesis methods

A typical laser ablation method can be employed for the removal of metallic ions from metal surfaces by using a laser beam and a small volume of liquid (methanol, ethanol, or purified water) in which the surface is immersed (Mintcheva et al. 2018). This approach offers the advantages of simplicity and environmental safety, making it an efficient and straightforward process. However, when organic substances are present, the pyrolysis byproducts resulting from laser ablation have not been fully elucidated and require further investigation. Noteworthy findings include those by Al-Dahash et al., who successfully employed laser ablation in a NaOH aqueous solution to produce spherical ZnO NPs ranging from 80.76 to 102.54 nm (Al-Dahash et al. 2018). Laser ablation offers advantages such as high precision, minimal heat-affected zones, and the ability to work with a wide range of materials. However, it also requires careful control of laser parameters to achieve desired results and avoid collateral damage to surrounding materials.

### Green syntheses

The green approach regarding the synthesis of ZnO NPs appeared as a result of the toxic substances used in the synthesis and the high energy consumption. Green synthesis or biosynthesis, as an alternative in obtaining NPs, involves the use of plant metabolites, microorganisms, and algae. Obtaining ZnO NPs, as an inorganic semiconductor, leads to the formation of amphoteric particles, insoluble in water (Hussein and Mohammed 2021).

There is numerous specialized literature that indicates various approaches regarding green processes, which can lead to the biosynthesis of nanoparticles through the use of plants and microorganisms (such as bacteria, fungi, algae) as an environmentally friendly, cost-effective, safe, byproduct-free, ecological solution (Agarwal et al. 2017; Salam et al. 2014). Those nanomaterials resulting from green synthesis using plants, microorganisms, algae, or other bioregenerable materials are considered “biogenic” (Jagpreet Singh et al. 2018; Prasad et al. 2021).

Green methods represent solutions for replacing hydrocarbon capping agents functionalized with heteroatoms, polymers (polyvinyl pyrrolidone, polyvinyl alcohol, etc.), dendrimers, and block copolymers with extracts from plants, fungi, yeasts, bacteria, and algae (Prasad et al. 2021; Duan et al. 2015; Prakash et al. 2010; Dauthal and Mukhopadhyay 2016; Dahoumane et al. 2017; Jha and Prasad 2008; Mukherjee et al. 2001). Thus, the replacement agents are polysaccharides (starch, chitosan, glucose, etc.), enzymes, polyphenols, vitamins, and biomolecules. The starch used in obtaining ZnO NPs, with the role of stabilizer, binds the metal ions from the precursor solution through the hydroxyl groups. The long polysaccharide chains reduce the mobility of metal ions and lead to an ordering of the structure, dimensions, and morphology of the synthesized nanoparticles (Mukherjee et al. 2001).

#### Plant extract synthesis method

Natural reagents have the role of reducers for nanoparticle precursors (usually salts), but they can also be stabilizing or coating agents. From certain parts of plants, from roots and fruits to seeds, reducing extracts and stabilizer properties for ZnO-type nanoparticles can be obtained, such as: *Trifolium pratense* flowers, *Aloe vera* leaves, or *Rosa canina* fruits (Zong et al. 2014; Ramesh et al. 2015). Green synthesis using plant extracts such as leaves, stems, roots, fruits, or seeds involves a fast synthesis time and leads to the production of a pure, stable material that can have various shapes and sizes (Agarwal et al. 2017; Jiao Qu et al. 2011b). The reduction of metal ions or oxides, using plant extracts, leads to the obtaining of 0 valence metal NPs, the reducing agent being polysaccharides, polyphenols, vitamins, amino acids, alkaloids, or terpenoids secreted by the plant (Jiao Qu et al. 2011b; Heinlaan et al. 2008).

The extract is prepared by boiling and stirring ground powder obtained from plant parts with demineralized water. After filtration, the resulting clarified solution serves as an extract that functions as a reducer for metal precursors (Heinlaan et al. 2008). In the case of ZnO NP preparation, hydrated zinc nitrate, zinc oxide, or zinc sulfate precursors can be mixed with the plant extract at the effective temperature and time (Ochieng et al. 2015; Jiao Qu et al. 2011a). The effectiveness of the extraction is greatly influenced by the temperature. In general, higher temperatures increase yields by increasing the rate of phytochemical diffusion and their solubility in the solvent. By lowering viscosity and surface tension, this also facilitates the penetration of the solvent into the plant matrix (Farahmandfar et al. 2019; Khan et al. 2019). However, the ideal temperature varies with respect to the plant, solvent, and targeted phytocompounds, and exceeding it can lead to the degradation of thermolabile biomolecules and an increase in solvent evaporation, which decreases efficiency. For instance, the amount of polyphenolic chemicals produced by *Orthosiphon stamineus* leaf extract peaked at 40 °C with 80% methanol and began to decline at 60 °C as a result of degradation (Akowuah and Zhari 2010). Similar to this, yields were decreased when anthocyanins were extracted from berries using ethanol or sulfured water above 45 °C (Cacace and Mazza 2003). In order to maximize the extraction of phytochemicals from various plant sources, proper temperature management is essential. The extraction yield can be affected by a number of factors, in addition to temperature, including stirring rate, extraction time, the solvent types and their ratio of mixing, particle size, and, of course, the method of extraction used. All of these factors must be considered during the extraction process (Sulaiman et al. 2017; Weldegebrieal 2020). Physical characteristics including particle size and shape (Stan et al. 2015), oxygen vacancy content (J. P. Wang et al. 2012b), and surface defects (edges and corners) can all have an impact on how effective ZnO NPs are as photocatalysts, as can the type of plant extract utilized and the synthesis procedure in general.

**Fig. 6:**
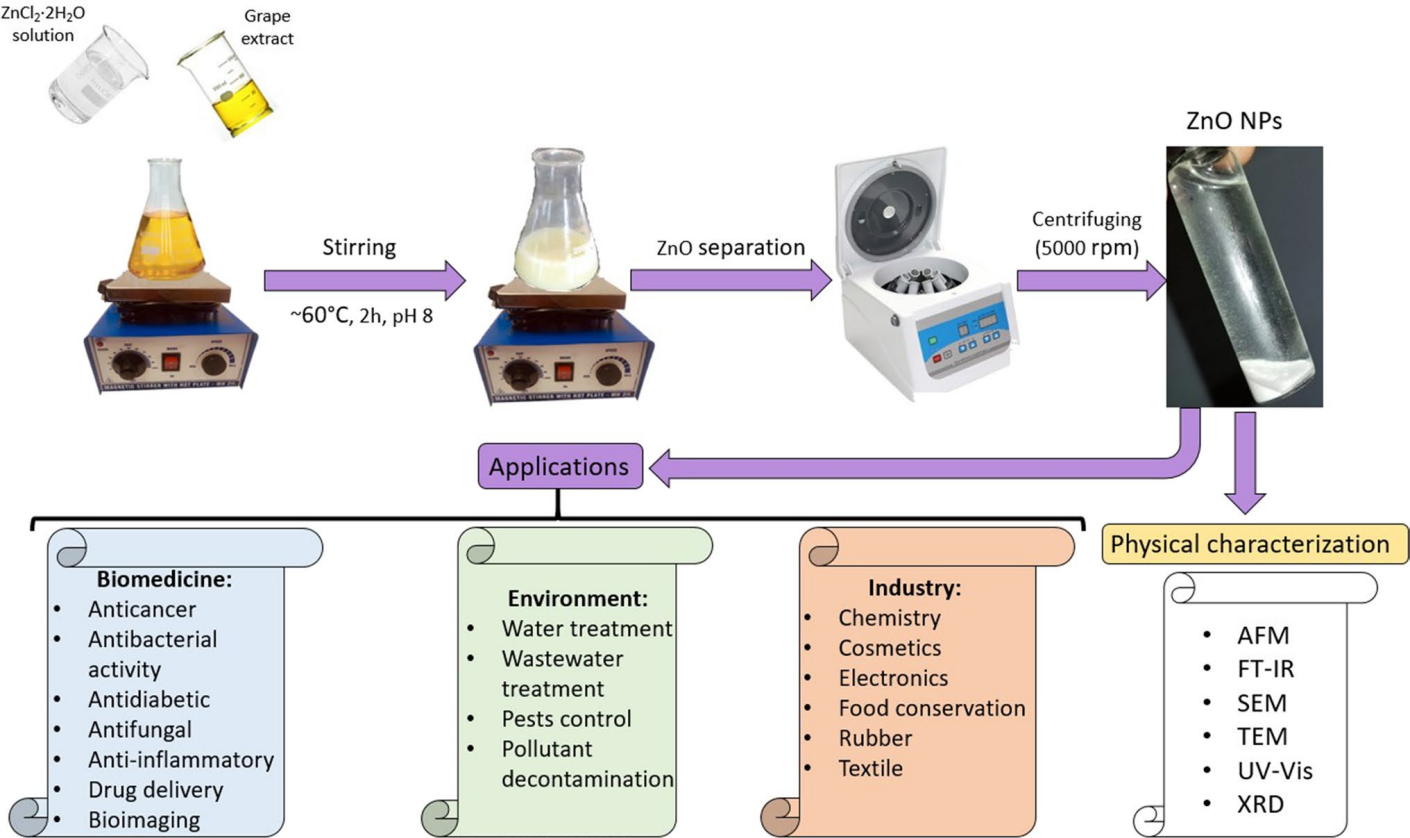
ZnO NP green synthesis and applications (AFM atomic force microscopy, FT-IR Fourier-transform infrared spectroscopy, SEM scanning electron microscopy, TEM transmission electron microscopy, UV–Vis ultraviolet–visible spectroscopy, XRD X-ray diffraction)

An example of a source of polyphenols is grape extract (*Vitis vinifera*), a source rich in phytochemicals, which also contains flavonoids and catechins, all with a reducing role for metal salts, but also other compounds of the nitro or ketone type (Pati et al. 2014; Khosravi-Darani et al. 2019; Upadhyay et al. 2015; Georgiev et al. 2014). ZnO NPs can be obtained from different zinc precursors (zinc chloride dihydrate, zinc sulfate heptahydrate ZnSO_4_ · 7H_2_O, etc.) solution and grape extract in the presence of NaOH to maintain the pH of the mixture at a value of 8, the white color indicating the formation of ZnO NPs (Fig. 6) (Constandache et al. 2023; Hussein and Mohammed 2021).

**Fig. 7:**
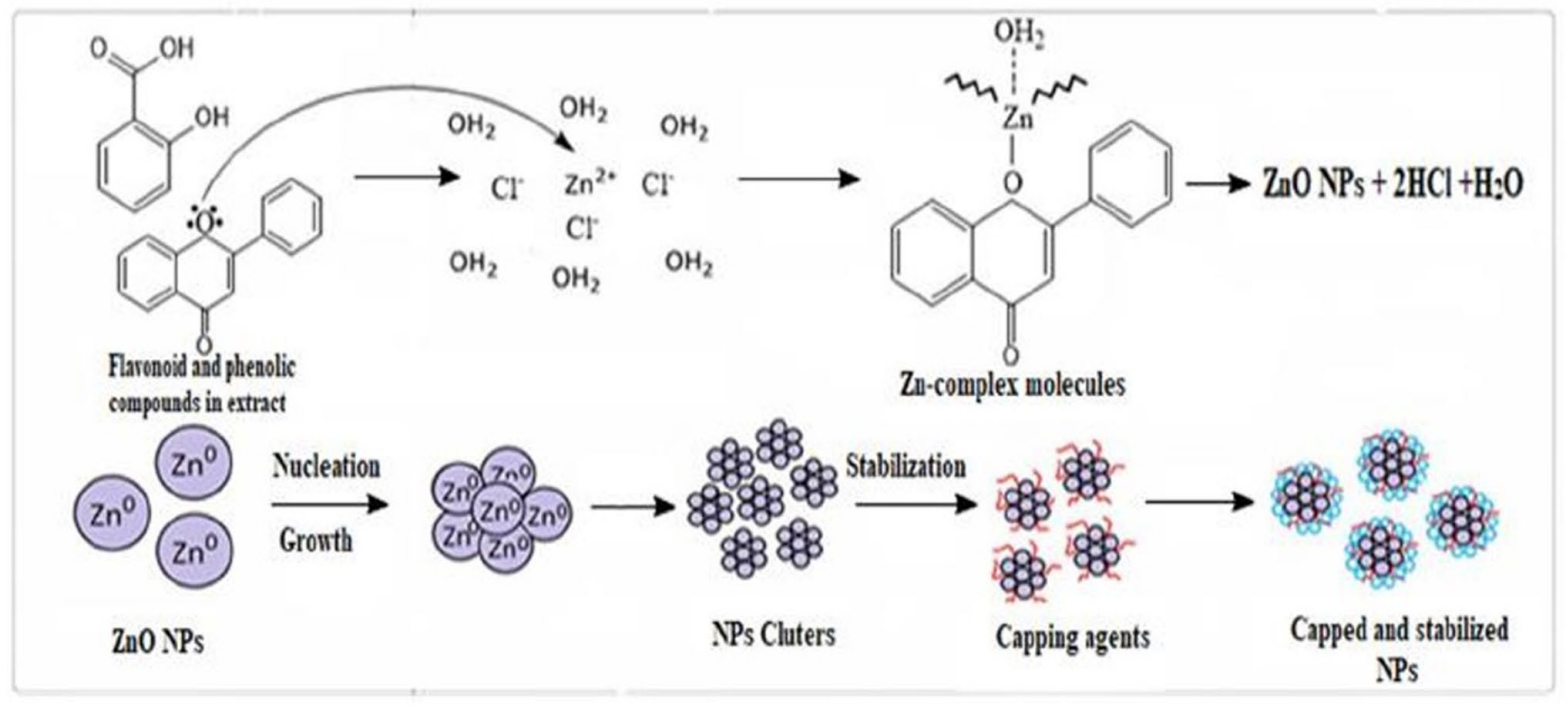
Mechanism of ZnO NP formation (permission) (Hussein and Mohammed 2021)

ZnO NPs are formed based on the reduction of the metal from the initial compound (e.g., salt) to zero valence, after which Zn oxide is formed by calcination. In the case of polyphenols from plant extracts, they produce complexation with Zn^2+^; by hydrolysis, Zn(OH)_2_ is formed, and by calcination, ZnO (Fig. 7). The formation mechanisms are not fully elucidated; they are still being studied (Basnet et al. 2018; Alamdari et al. 2020; Barzinjy et al. 2020).

One of the advantages of green synthesis is the integration of ZnO NP synthesis processes into the concept of sustainability and waste minimization (Barzinjy et al. 2020). Physicochemical methods are still applied due to the short synthesis time, although the equipment is energy consuming, and the reagents include acids and bases, surfactants, and solvents with toxic, corrosive, and irritating potential. It also results in hazardous waste and toxic gases (Barzinjy et al. 2020). The ecological methods of synthesis also involve the reuse of some vegetable waste, a potential source of phytochemical substances in the processes of obtaining ZnO NPs.

The literature indicates the use of *Carica papaya* extracts and the one obtained from *Vitex negundo* leaves on the basis of which possible stages were developed in the mechanism of obtaining ZnO NPs: complexation, aggregation of NPs, and oxidation of phytocomponents (SC Sharma 2016; Ambika and Sundrarajan 2015).

Other types of plant extracts through which the presence of polyols, phenolic acids, flavonoids, sugars, tannins, and acids leads to the obtaining of stable and controlled ZnO NPs are those obtained from the leaf of *Swertia chirayita*, fruits of *Rosa canina*, bark of *Boswellia* stem, *mimosifolia* flower, *Camellia sinensis* tea leaves, *Plectranthus amboinicus* leaves, etc. (Prasad et al. 2021; Akhter et al. 2018; Jafarirad et al. 2016; Supraja et al. 2016; Vijayakumar et al. 2015; Senthilkumar and Sivakumar 2014; Deepali Sharma et al. 2016).

The citrus extract, especially lemon, led to the obtaining of ZnO NPs, using dry and crushed leaves dissolved in deionized water, the obtained filtrate being brought into contact with a precursor solution of Zn(NO_3_)_2_·6H_2_O, in the presence of a solution of NaOH. After maintaining the temperature for 3 h and drying the precipitate, ZnO NPs of approx. 15–25 nm were obtained (Karanpal Singh et al. 2023).

Today, green synthesis is integrated into laboratory synthesis methods in which nanocomposites can be obtained from natural precursors. Thus, the extract from crushed leaves of *Ageratum houstonianum* was brought into contact with Zn(NO_3_)_2_·6H_2_O solution, the precipitate formed being dried in an oven at 60 °C and calcined at 700 °C for obtaining crystalline ZnO NPs. In order to make the dye degradation process more efficient by irradiation with natural sunlight, NPs were anchored in multilayer graphene obtained from corn husk, thus obtaining nanocomposites with a crystallite size of about 40 nm (Sebuso et al. 2022).

Recently, significant interest in template-assisted environmentally friendly synthetic methods has been shown, which are cost-effective and safe by avoiding the use of organic solvents, surfactants, and hazardous chemicals. Thus, ZnO nanostructures synthesized with the assistance of xanthan gum (XG) were created using three different green protocols: sonochemical, mechanochemical, and hydrothermal methods (Kaur et al. 2023). These ZnO nanostructures were denoted as ZnO-TS, ZnO-TH, and ZnO-TM, respectively. Similarly, ZnO was synthesized without the use of XG as a template through sonochemical, hydrothermal, and mechanochemical methods, and these resultant ZnO samples were labeled as ZnO-S, ZnO-H, and ZnO-M, respectively. The template-assisted ZnO nanostructures were assessed for their potential as photocatalysts in the degradation of emerging pollutants, specifically triclosan (TCS) and imidacloprid (IMD), under UV light exposure and revealed high photocatalytic performance. It was reported that the photocatalytic efficiency of the catalyst was significantly influenced by several factors, including crystallite size, surface area, and the band gap energy of the catalyst. Due to its smaller crystallite size, larger surface area, and lower band gap energy, the ZnO-TS photocatalyst outperformed both the ZnO-TH and ZnO-TM catalysts in terms of catalytic efficiency, particularly under UV light.

**Fig. 8:**
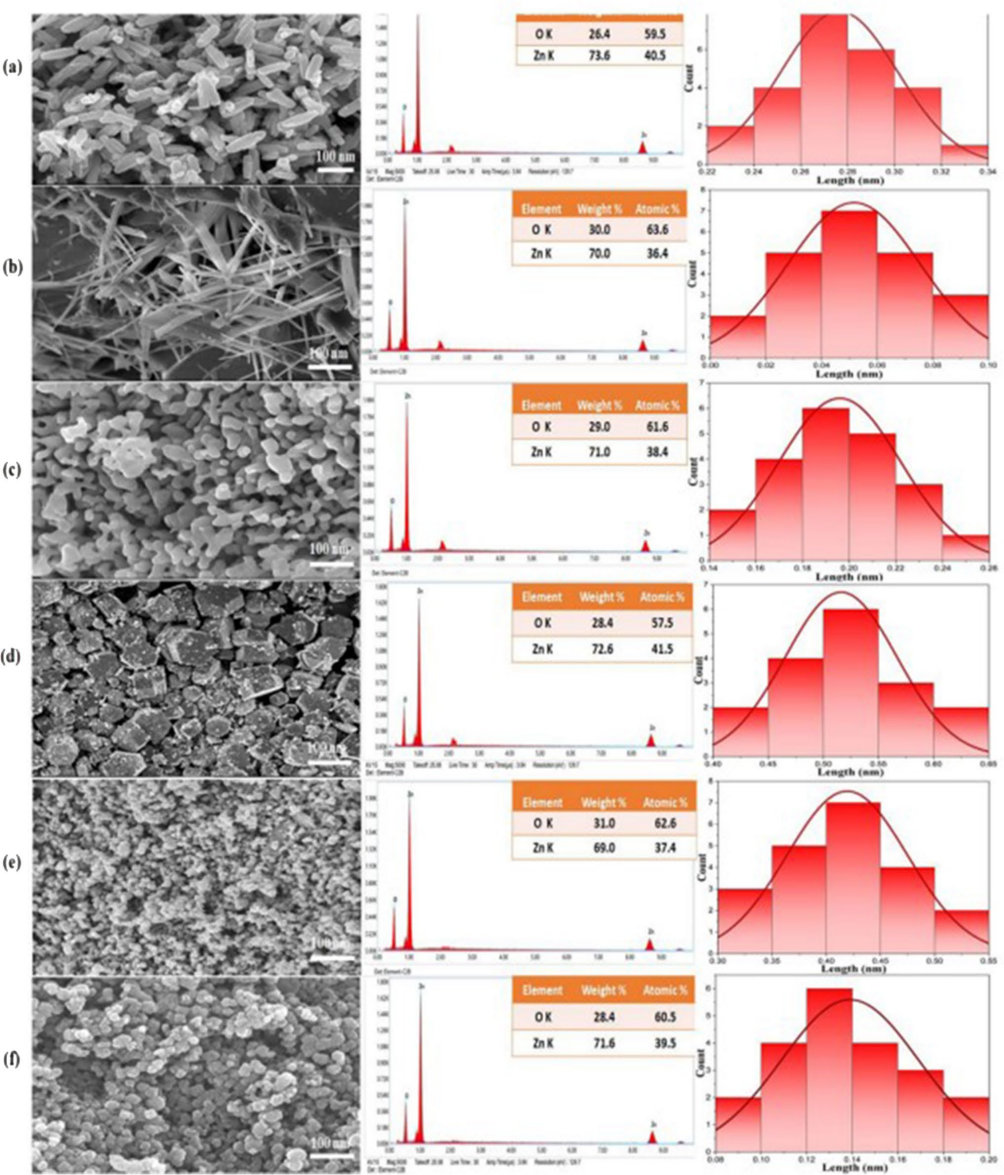
FESEM images, EDS, and particle size distribution curve of **a** ZnO-S, **b** ZnO-TS, **c** ZnO-H, **d** ZnO-TH, **e** ZnO-M, and **f** ZnO-TM nanostructures (with permission from Kaur et al. 2023)

The analysis conducted through field emission scanning electron microscopy (FESEM) demonstrated that the synthesis methods had a substantial influence on the morphology of ZnO, resulting in various nanostructures depending on the chosen method (Fig. 8). Specifically, the template-free sonochemical synthesis approach (ZnO-S sample) yielded rod-like ZnO structures, while the template-assisted sonochemical synthesis (ZnO-TS) produced elongated needle-shaped particles. In the case of hydrothermal synthesis, both template-assisted (ZnO-TH) and template-free (ZnO-H) methods produced distinct morphologies: highly crystalline hexagonal and worm-like shapes, respectively (Kaur et al. 2023).

A different research paper documented the utilization of fresh olive (*Olea europaea*) fruit extract in the synthesis of zinc oxide NPs (ZnO@OFE NPs) (Ghaffar et al. 2023). These nanoparticles exhibited a spherical nanostructure with a diameter of 57 nm and were produced through an eco-friendly one-pot method. Waste from *O. europaea* fruit played a dual role as a reducing agent and capping agent in this process. The effectiveness of the newly synthesized catalysts was assessed by observing the degradation of methylene blue (MB) and methyl orange (MO) dyes when exposed to sunlight. To conduct a comprehensive analysis of their photocatalytic activity, a catalyst dose of 30 mg in 30 mL of solution was determined to provide optimal absorptions of 75% for MB and 87% for MO within 180 min, with photodegradation rate constants of 0.008 and 0.013 min^−1^, respectively. Additionally, the ZnO@OFE NPs displayed impressive antioxidant properties, combating DPPH, hydroxyl, peroxide, and superoxide radicals. In contrast, when sunlight was absent, the removal efficiency via adsorption onto the ZnO@OFE NPs’ surface was only 12% for MB dye and 10% for MO dye after 180 min.

Bimetallic ZnO–CuO hetero-nanocomposite, ZnO, and CuO nanostructures were synthesized utilizing a hydrothermal synthetic procedure, employing leaf extract from *Aegle marmelos*, also known as bael (Basavegowda et al. 2022). The ZnO–CuO hetero-nanocomposite and ZnO NPs were found to be spherical, with an average size of approximately 9.2 nm and 7.8 nm, respectively. The high stability of the ZnO–CuO hetero-nanocomposite, ZnO, and CuO NPs in an aqueous medium was confirmed by ζ-potential measurements, which were recorded as − 28.2, − 33.2, and − 29.6 mV, respectively. Benefiting from the formation of p-n heterostructures, the bimetallic ZnO–CuO hetero-nanocomposite exhibited excellent photocatalytic activity against 4-nitroaniline (4-NA) and MO compared to pure ZnO and CuO. The photocatalytic performance results showed a significant improvement for the bimetallic ZnO–CuO hetero-nanocomposite in degrading 4-NA (90% removal in 20 min, with a rate constant, *k*, of 3.9 × 10–2 min^−1^) and MO (96% removal in 10 min, with a *k* of 41.15 × 10–2 min^−1^). These outcomes were twofold compared to the performance of pure ZnO and CuO NPs.

#### Bacteria synthesis method

Green synthesis of ZnO NPs can also be achieved using bacteria, although this requires the screening for microbes and a careful monitoring of the culture broth, resulting in additional costs and extended processing times. Some bacterial strains have the capacity to reduce metal ions leading to the synthesis of oxide nanoparticles: *Lactobacillus casei*, prokaryotic bacteria, actinomycetes, *Escherichia coli*, etc. Yeasts can be successfully applied in green synthesis to obtain nanoparticles of silver, gold, zinc, and/or titanium (Iravani 2014; Thakkar et al. 2010). Intracellular enzymes present in microorganisms have the role of reducing metal ions until the formation of NPs. Green synthesis brings together two areas of interest for the progress of society, materials science and environmental protection, through which nanomaterials gain a sustainable path according to their life cycle.

Utilizing bacteria to create nanoparticles comes with various challenges, such as the considerable time and effort needed to identify suitable microorganisms, the requirement for continuous monitoring throughout the culture and synthesis process, and concerns related to controlling the shape and size of the nanoparticles. Additionally, the expense associated with the growth media for bacteria can act as a limiting factor. Nevertheless, an environmentally friendly approach was demonstrated by utilizing *Bacillus licheniformis* to produce ZnO nanoflowers. These nanoflowers showcased an improved level of photocatalytic activity and degradation capabilities when compared to existing photocatalytic materials. This enhancement is attributed to the higher concentration of oxygen vacancies within the synthesized nanoparticles. These distinct photocatalytic characteristics suggest that these nanoflowers hold potential for applications in bioremediation processes, as they generate active species through the absorption of light. The nanoflowers produced using this method had dimensions of approximately 40 nm in width and 400 nm in length (Raliya and Tarafdar 2013).

A study by Tripathi et al. leveraged the unique capabilities of *Rhodococcus* and *Aeromonas hydrophila* to synthesize ZnO NPs of different sizes and shapes (Tripathi et al. 2014). The study also highlighted the role of rhamnolipid in stabilizing these NPs, showcasing their potential for various applications in nanotechnology and materials science. *Rhodococcus*, known for its resilience in adverse conditions and its ability to metabolize hydrophobic substances, plays a crucial role in biodegradation (Reddy et al. 2012). In this study, *Rhodococcus pyridinivorans* and zinc sulfate were utilized to produce spherical NPs with a size range of 100–130 nm, as confirmed by XRD and FESEM analysis. FTIR examination identified various chemical groups present in the NPs. Additionally, ZnO NPs were synthesized using *Aeromonas hydrophila* as a substrate, resulting in NPs with a size range of 42–64 nm, featuring diverse shapes such as oval and spherical (Mehta et al. 2009). Rhamnolipid, due to its ability to prevent micelle aggregation on carboxymethyl cellulose, contributed to the stability of these ZnO NPs, acting as an effective capping agent (Kundu et al. 2014). Further characterization through TEM, XRD, and DLS revealed the synthesis of spherical NPs with a nanosize range of 27–81 nm.

#### Micro- and macroalgae synthesis method

Unicellular algae (like *chlorella*) and multicellular algae (such as *chlorophyll*) serve as examples of photosynthetic organisms, notably including brown algae. Unlike conventional plants, algae lack typical plant structures like leaves and roots. Marine algae are categorized based on their pigments, with Rhodophyta, Phaeophyta, and chlorophytes characterized by red, brown, and green pigments, respectively. Algae have been extensively utilized for producing gold (Au) and silver (Ag) nanoparticles, but their application in synthesizing zinc oxide (ZnO) nanoparticles has been limited and is documented in relatively few studies (Thema et al. 2015).

Significant attention has been directed towards the potential of microalgae to detoxify hazardous metals and convert them into less harmful forms. To synthesize ZnO NPs, researchers have employed both *Sargassum muticum* and *Sargassum myriocystum*, which are both part of the *Sargassaceae* plant family (Sanaeimehr et al. 2018; Azizi et al. 2014). The analysis using XRD and FESEM revealed the presence of sulfated polysaccharides in the investigated NPs, indicating similar nanoparticle sizes (42 nm, 30–57 nm, respectively) and a hexagonal wurtzite structure. In the case of *Sargassum myriocystum*, DLS and AFM measurements showed varying size ranges (46.6 nm, 20–36 nm, respectively), along with the identification of carbonyl and hydroxyl stretching in nanoparticles that exhibited substantial structural diversity. From some microalgae, ZnO NPs of about 36 nm can be obtained, whose stability has been demonstrated through structural investigations even after 6 months (Agarwal et al. 2017).

An eco-friendly, biologically mediated method for producing ZnO nanoflowers at low temperatures has been investigated in a recent study (Rao and Gautam 2016). This “green” strategy has a number of benefits, including the use of environmentally benign reactants and financial viability. These nanoflowers were developed by the researchers from the cell-free extract of the freshwater microalga *Chlamydomonas reinhardtii* and were composed of individual nanorods assembling into flower-like shapes. The 330-nm-long nanorods created porous nanosheets that were 55–80 nm thick. The size of larger porous nanoflowers was about 4 µm. The ZnO nanoflowers’ hexagonal wurtzite crystal structure was confirmed by XRD analysis, and algal biomolecules may have contributed to their formation and stabilization, according to FTIR spectroscopy. The effects of dye concentration and catalyst dose revealed that these nanoflowers demonstrated improved photocatalytic activity against methyl orange (MO) under natural sunshine. This technology offers a cutting-edge, environmentally benign way to make zinc oxide nanoflowers with potential uses in water purification and dye deterioration.

#### Fungus synthesis method

An affordable and cost-effective alternative is the use of fungi in the production of ZnO NPs, as they exhibit tolerance and bioaccumulation of metals compared to bacteria (Pati et al. 2014). Extracellular NPs from fungus are advantageous due to their high production, simple downstream processing, and commercial viability (Azizi et al. 2014).

ZnO NPs were made using *Aspergillus fumigatus* mycelia. The DLS study found that the area sizes of NPs ranged from 1.2 to 6.8, with a 3.8 average size. With a considerable particle size of more than 100 nm, AFM determined that the average height of NPs for a period of 90 days was 8.56 nm. The generated NPs were stable for 90 days because they eventually formed an agglomeration with an average particle size of 100 nm (Jaidev and Narasimha 2010). The NPs made from *Aspergillus terreus*, a member of the *Trichocomaceae* family, were subjected to SEM analyses and the size range was between 54.8 and 82.6 nm. The Debye–Scherrer equation was used to calculate the average size of the material found in the XRD analysis, which was 29 nm. In the created NPs, FTIR measurements revealed the synthesis of primary alcohol, aromatic nitro compounds, and amine. Analysis using SEM, TEM, and XRD confirmed that NPs made with *Candida albicans* had a similar size range of 15–25 nm (Shamsuzzaman et al. 2017). ZnO NPs produced by *Aspergillus* species were typically spherical.

Another study presented the synthesis of ZnO NPs by using a fungal extract from *Xylaria acuta* and the obtained nanopowder was characterized by means of UV spectroscopy, FT-IR, PXRD, SEM with EDX, and TEM to determine the structure, morphology, and chemical composition (Sumanth et al. 2020). The SEM analyses showed that the structures of the NPs were cylindrical rods and hexagonal shapes with an average diameter of 40–55 nm. To determine the elemental composition and existence of the ZnO NPs, an EDX scan was performed. Zinc and oxygen showed prominent signals in the EDX spectrum, which verified the existence of ZnO NPs produced by fungi and the fact that zinc exists as an oxide as opposed to its pure form (Shankar and Rhim 2019).

*Aspergillus niger* can effectively manufacture ZnO NPs on a wide scale, as shown by a study’s characterization using XRD, UV–Vis, FTIR, and SEM (Kalpana et al. 2018). The functional groups present in the NPs were investigated with FTIR analysis. The effective synthesis of ZnO NPs is demonstrated by distinct and powerful diffraction peaks in the XRD examination. SEM has verified that these NPs are spherical and range in size from 84 to 91 nm. The research also shows that cotton fabric treated with these nano-ZnO NPs has antibacterial qualities. Furthermore, a decrease in absorbance, indicating complete mineralization and color loss, showed that the synthesized NPs were successful in degrading Bismarck brown dye.

## Recent contributions regarding emerging pollutant (EP) degradation towards photocatalysis

### Impact of the EPs on the environment

**Fig. 9:**
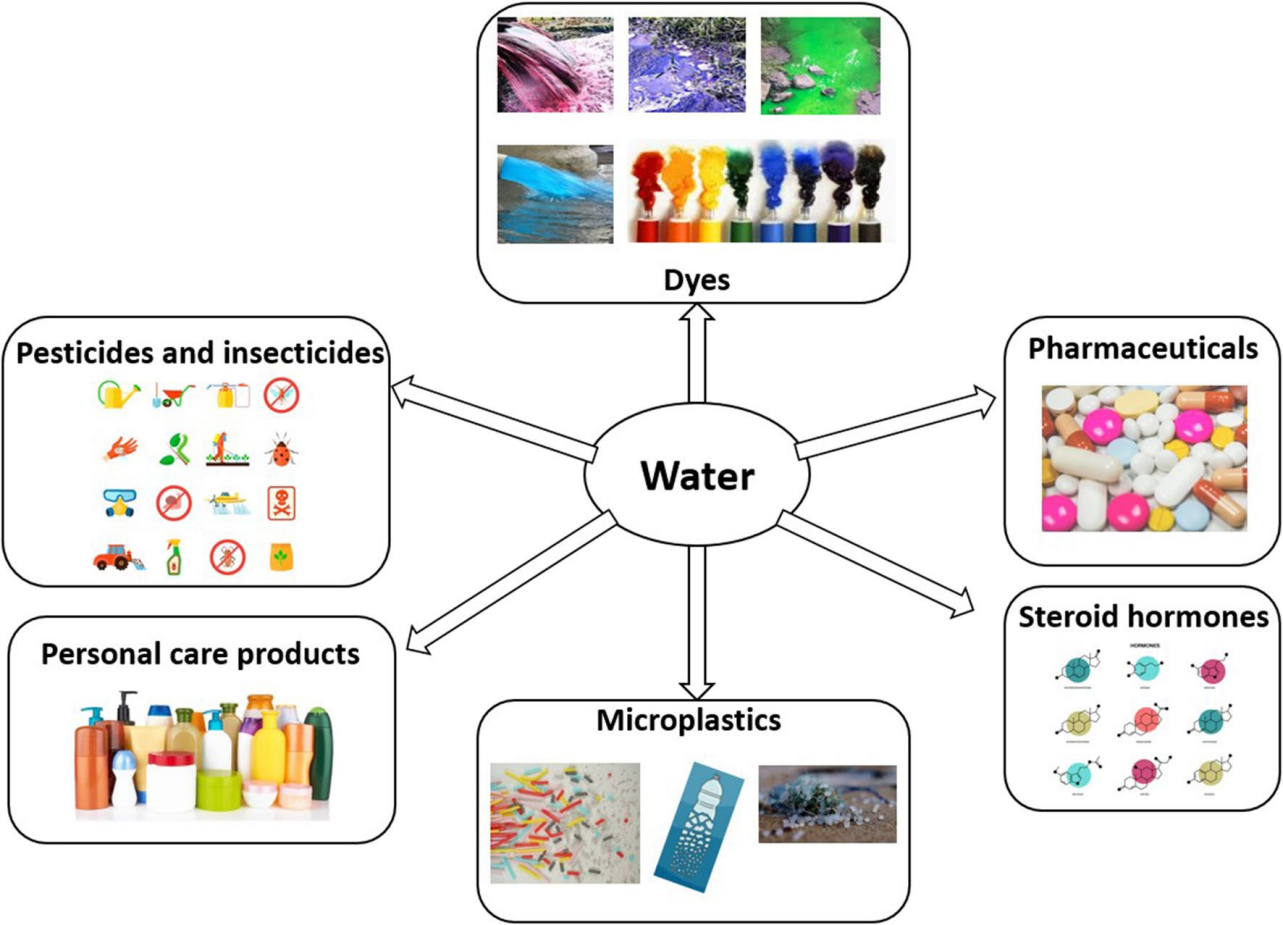
Most common categories of emerging pollutants present in water

EPs are a new class of substances that at low concentrations are identified as presenting ecological and human health risks (Guanqun Feng et al. 2021; Noguera-Oviedo and Aga 2016). EPs comprise few subgroups of organics: pharmaceutical and personal care products (PPCP), microplastics (MP), engineered nanomaterials (ENM), pharmaceutically active compounds (PhAC), endocrine-disrupting chemicals (EDC), artificial sweeteners (ASW), disinfection byproducts (DBP), antibiotic resistance genes (ARG), detergents, pesticides, and other organic compounds that are mainly generated by human activities (Noguera-Oviedo and Aga 2016; Qiaowen Tan et al. 2019a; Zhang et al. 2020; Pruden et al. 2006). A comprehensive illustration of the most common categories of emerging pollutants presented in water is shown in Fig. 9.

Konstantinou and Albanis observed that textile dyes and other industrial dye compounds form a substantial group of organic substances posing an increasing environmental risk (Konstantinou and Albanis 2004). The discharge of colored wastewater into the environment causes eutrophication and non-esthetic pollution, and it may produce harmful byproducts due to chemical reactions occurring within the effluent. These dyes can also be toxic, which heightens ecological concerns about their presence in the environment. They can also make it harder for light to penetrate contaminated waterways (Akpan and Hameed 2009). Traditional wastewater treatment methods have proven ineffective in treating synthetic textile dye wastewater due to the dyes’ chemical stability, with some dyes passing through untreated. Furthermore, textile dyes are resistant to conventional methods of degradation. To address this issue, recent research has focused on photocatalysis as a promising approach for completely breaking down these pollutants in wastewater (Saquib et al. 2008; Weldegebrieal 2020; Moradi et al. 2015).

Pharmaceuticals and personal care products constitute a unique category of substances, often categorized as emerging environmental pollutants due to their inherent ability to induce various physiological responses in humans. Many research inquiries have validated the presence of PPCPs in various environmental settings, raising concerns about potential significant consequences for both biodiversity and human well-being. Pharmaceuticals refer to medicinal substances, including over-the-counter and prescription drugs, used for treating and preventing illnesses in both humans and animals. On the other hand, personal care products are primarily designed to enhance our daily lives’ quality (Osuoha et al. 2023).

**Fig. 10:**
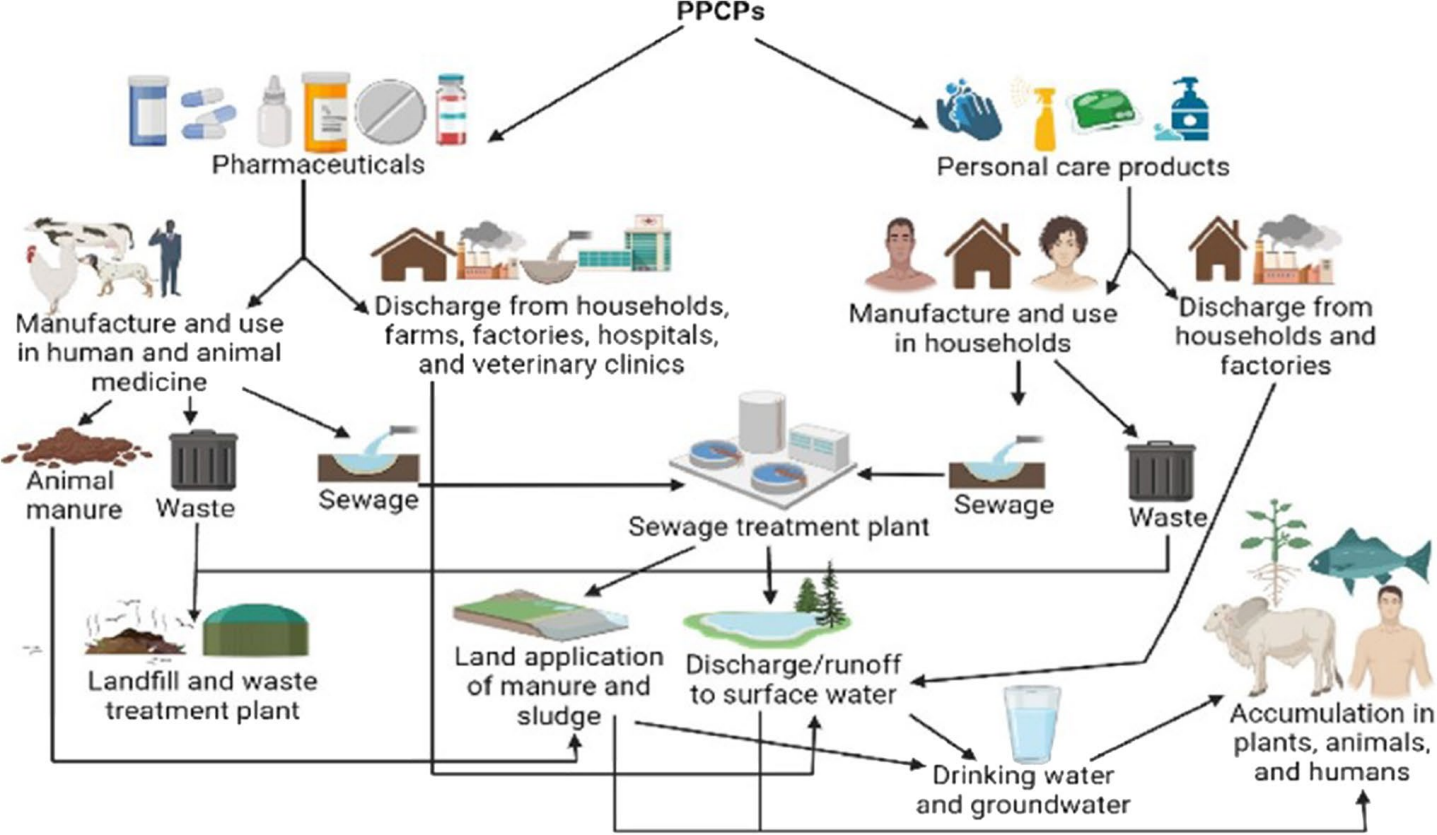
Sources and transportation of PPCPs in the environment (with permission from Osuoha et al. 2023)

PPCPs represent a substantial category of emerging pollutants. Over the last 10 years, significant apprehension has arisen due to the inadvertent presence of PPCPs in various components of the marine environment, including biota, sediments, and water, at concentrations with the potential to trigger adverse consequences for the immediate ecosystem. When administered in small amounts, most PPCPs exhibit a remarkable ability to induce physiological abnormalities, classifying them as potent substances capable of disrupting biological processes in a variety of organisms. Although certain PPCPs may undergo degradation in the environment, their persistent consumption and inadvertent introduction into ecosystems classify them as “pseudo-persistent” compounds within the environment. The main sources and transportation modes of PPCPs in the environment are presented in Fig. 10.

Many inquiries have been conducted concerning the prevalence, ecological harm, and methods of breaking down PPCPs in wastewater for their elimination. Although the research community has shown growing interest in PPCPs, there is still a significant gap when it comes to understanding their ecological impact. This gap is primarily due to the limited number of substances being studied across various environmental matrices. Some researchers have examined the concentrations of certain PPCPs, which are classified as priority chemicals in aquatic ecosystems (N. Liu et al. 2020; Junaid et al. 2019; Paucar et al. 2019; Yi Chen et al. 2016).

In a research conducted by Madikizela et al. in 2020 (Madikizela et al. 2020), it was documented that there was a presence of 19.2 μg L^−1^ of ibuprofen detected in surface water. Conversely, Matongo et al. (Matongo et al. 2015) observed a concentration of the same pharmaceutical and personal care product (PPCP) at 1.38 μg L^−1^ in wastewater. The dieldrin concentration was recorded at 1.51 μg L^−1^ in surface water, according to Okoya et al. (Okoya et al. 2013). Additionally, the levels of acetaminophen and amoxicillin in surface water and seawater ranged from 0.0058 to 1.23 μg L^−1^, as reported by Folarin et al. in 2020 (Folarin et al. 2019). Moreover, in a study conducted by Olatunde et al. in 2014, oxytetracycline was found to have concentrations ranging from 0.003 to 0.0048 μg L^−1^ in surface water (James et al. 2014). Similarly, Amdany et al. (2014) reported that naproxen, ibuprofen, and triclosan exhibited concentrations in wastewater ranging from 10.7 to 127.7 μg L^−1^. In wastewater, naproxen, ibuprofen, and triclosan were found to have concentrations ranging from 10.7 to 127.7 μg L^−1^ (Amdany et al. 2014). Nevertheless, the influence of sediment on the concentration and behavior of PPCPs in aquatic systems remains uncertain.

The concentrations of EPs are related to habits patterns, water consumption, sewer conditions, environmental fate, etc. (Parida et al. 2021). Even if the concentrations are between nanograms per liter and micrograms per liter, the risks regarding long-term exposure and environmental persistence could affect life and ecosystem’s health and sustainability, especially when inadequate treatment is applied (Taoufik et al. 2020; Parida et al. 2021; Lutterbeck et al. 2020; Yi Chen et al. 2016). Today, some international regulations stated the status of these EPs and developed lists with compounds classes, characteristics, and exposure information (Recast 2010; Post 2021; Organization and WHO., 2004).

Different treatment combinations are used today because the conventional treatment processes are ineffective for effectively removing EPs. Examples include biological treatment methods combined with AOP (e.g., membrane bioreactor-ozonation, constructed wetlands-UV irradiation) and membrane separation processes combined with biological treatments (e.g., membrane bioreactor-reverse osmosis, sequential biological reactor-nanofiltration) (Parida et al. 2021). The main constraints are given by the high costs and sustainability of processes. Thus, research is focusing on the development of new materials with cost-effective environmental efficiencies.

There are various benefits of using AOP for wastewater treatment. First off, because of the high oxidation potential of the ∙OH molecule (Kokkinos et al. 2021), AOPs are renowned for their quick reaction speeds. When compared to traditional treatment procedures, this speedy response results in shorter retention times, which improves the effectiveness of wastewater treatment. Second, AOP systems have a compact footprint that requires little space on the ground to process the required flow rate.

The limited introduction of new dangerous compounds into the water is a key advantage as well. ∙OH molecules can unite to form water, lowering the possibility of dangerous consequences, as opposed to chlorine disinfection, which might result in toxic byproducts. AOPs can also mineralize organic molecules, transforming them into stable inorganic substances including salts, water, and carbon dioxide. These methods are adaptable and able to remove a variety of organic materials as well as get rid of some heavy metals (Saviano et al. 2023). AOPs can successfully eliminate pathogens as a disinfection step when used in conjunction with UV disinfection, in particular. AOPs also do not concentrate trash for subsequent treatment, unlike chemical or biological processes, and do not produce sludge, which lowers the concentration of pollutants in the effluent (Deng and Zhao 2015).

ZnO NPs exhibit strong antibacterial properties, making them a promising candidate for various antimicrobial applications. The effectiveness of ZnO NPs against bacterial strains like *Bacillus subtilis* and *Escherichia coli* is attributed to their nanoscale size, which enhances their interactions with bacteria (Karanpal Singh et al. 2023). When reduced to the nanometer range, ZnO NPs can efficiently target bacterial surfaces and even penetrate inside the bacterial cells, leading to distinct bactericidal mechanisms (Seil and Webster 2012). These interactions are generally toxic to bacteria, making ZnO NPs valuable in antimicrobial applications, such as in the food industry and wastewater treatment. Moreover, their biocompatibility and thermal stability further enhance their potential as antibacterial agents (Karanpal Singh et al. 2023). Overall, the literature suggests that ZnO NPs possess a strong antimicrobial capability that can be harnessed for various biotechnological and environmental purposes.

One of the processes that lead to the removal of EPs, especially for antibiotics, is adsorption, which can be physisorption or chemisorption, and based on kinetic models and spectroscopic analyses, the correct mechanisms can be identified. The chemistry of the solution (pH, pKa, and pHpzc) is essential in establishing the interactions between adsorbate and adsorbent at different pH values (Ajala et al. 2022). The pH has an essential role in the photocatalytic degradation, its values influencing the surface charge of the catalyst, the method of binding the pollutants to the catalyst surface, and their dissociation (Adeola et al. 2022).

According to the literature, PPCP include any product used resulting from medical applications and care products, which appears in water and represents a health risk. The sources of release in the aquatic environment are multiple, and their appearance in water purification and treatment plants has started to represent a concern in the last 30 years (Schumock et al. 2014; Leung et al. 2012; Jin-Lin Liu and Wong 2013; Kosma et al. 2010; Oliveira et al. 2015; Ling Feng et al. 2013). The problem regarding their persistence is given by the concentrations and stability of the initial structures, in water they can be transformed and immobilized.

The stability of EPs to oxidation treatments and their toxicity make their biological degradation impossible and thus, their presence in the environment is a major risk. An example is acetaminophen (ACE), which ends up in water through urine, after consumption (Tobajas et al. 2017). High concentrations can lead to liver diseases, especially through decomposition products (Roberts and Thomas 2006; Buxton and Kolpin 2005; Ternes 1998). Another example is antipyrine (ANT) which can cause liver damage in case of uncontrolled occurrence in aquatic environments, this being detected in a proportion of 68.5% after treatment with activated sludge (Deblonde et al. 2011).

**Fig. 11:**
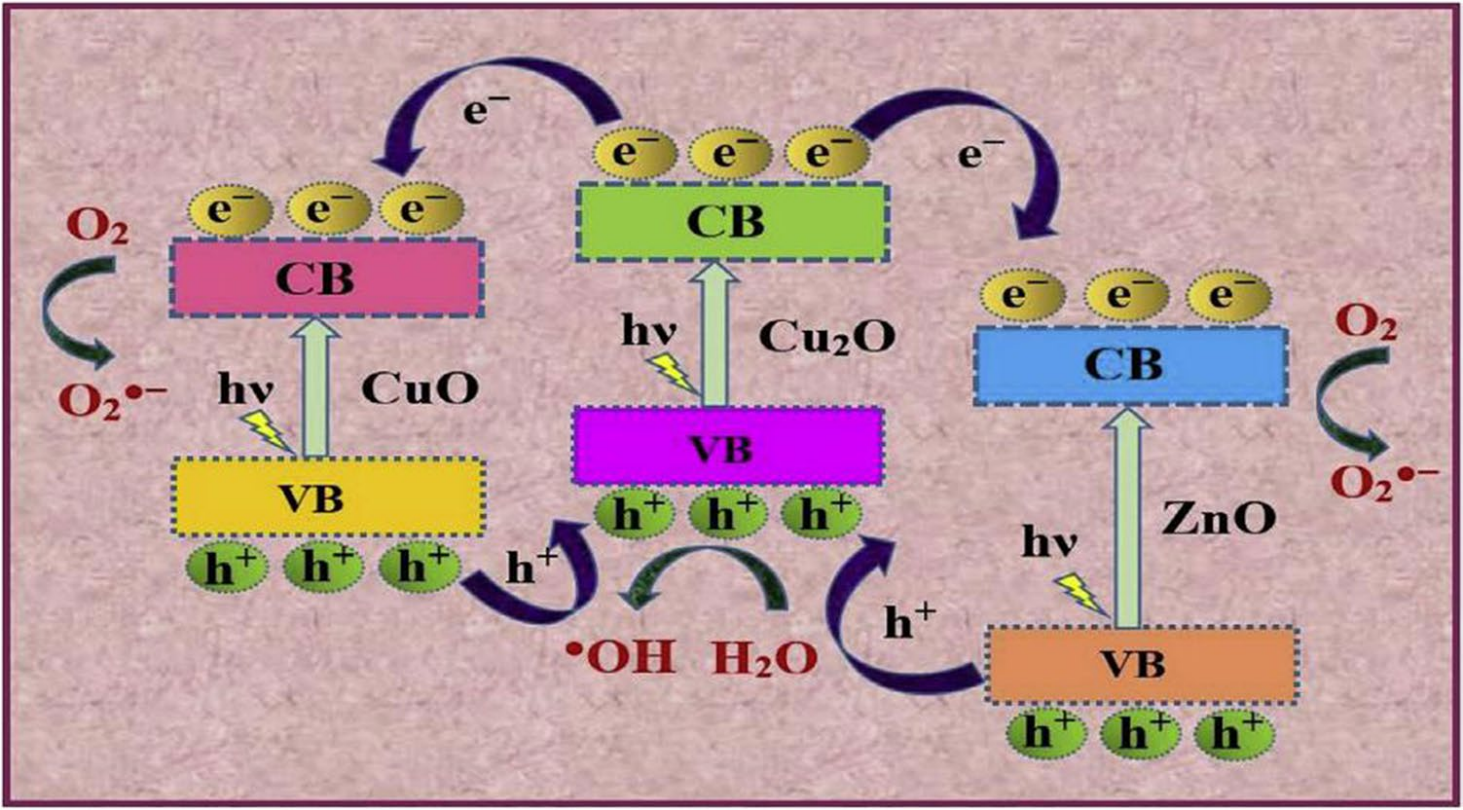
Charge transfer mechanism in ZnO**-**Cu_*x*_O heterostructure. CV conduction band, VB valence band (with permission from Adeola et al. 2022)

The photocatalysis process follows specific kinetic models as indicated in the literature, which serves as the basis for establishing the degradation mechanism. Adeola et al. indicate the first-order Langmuir–Hinshelwood model for the ZnO-Cu_*x*_O photocatalyst (Adeola et al. 2022). The mechanism is due to superoxide as the main generator of reactive species, and holes (h^+^) lead to the formation of reactive oxygen species (ROS), such as hydroxyl radicals (OH^∙^) and superoxide radicals (O_2_^∙−^). These ROS are highly reactive and can attack and degrade the pollutants, further enhancing the photocatalytic activity of ZnO. The degradation process that was based on this mechanism was successfully applied in the degradation of dyes and is presented in Fig. 11.

### Photodegradation performances of ZnO nanostructures applied for EP degradation

An efficient photocatalyst must provide good charge separation and efficient light absorption, especially in the visible region. Thus, semiconductors alone require a high intensity of light, induce charge carriers, and thus lead to limitations of their applicability. Doping with elements or obtaining a composite photocatalyst can provide solutions to these challenges (Shoaib Ahmed et al. 2021; Taoufik et al. 2020; Parida et al. 2021). It is necessary to obtain materials with a narrow band gap, low charge recombination rate, visible light irradiation, reusability, and stability. A few examples of the photodegradation efficiency (PE %) of some recently used photocatalysts based on ZnO NPs for emerging pollutant degradation are presented in Table 1.

**Table 1:** Photocatalysts used for degradation of emerging pollutants in water and wastewater

Photocatalyst	Organic pollutants	Optimum conditions	PE (%)	Ref
rGO/TIO_2_/ZnO	BPA Ibuprofen (IBP) Flurbiprofen	pH 5, initial conc. 10 mg/L, catalyst dose 0.025 g, reaction time 3 h	94.9 79.6 82.2	(Bilgin Simsek et al. 2018)
PGCN/AgI/ZnO/CQDs	2–4-Dinitrophenol	pH 4, initial conc. 10 mg/L, catalyst dose 50 mg, reaction time 140 min	> 90	(Hasija et al. 2019)
C/ZnO/CdS	4-Chlorophenol	Initial conc. 10 mg/L, catalyst dose 0.05 g, reaction time 120 min	98	(Atul B. Lavand and Malghe 2015b)
Au/-Pd-TiO_2_-ZnO	Malathion	Initial conc. 10 mg/L, catalyst dose 0.05 g, reaction time 120 min	98.2	(Vaya and Surolia 2020)
ZnO/SnO_2_	MB	Initial conc. 20 mg/L, catalyst dose 0.2 g/L, reaction time 60 min	97	(Lin et al. 2018)
Fe_3_O_4_@rGO@ZnO@Ag NPs	Metformin	pH 5.4, Initial conc. 20 mg/L, catalyst dose 1 g/L, reaction time 60 min	100	(Khavar et al. 2019)
N-doped ZnO	Tetracycline	30 ppm, Xe lamp with glass filter (300 W); dose 300 mg/L; time 2 h	97	(Xiao Chen et al. 2020b)
Ag_2_O/ZnO/rGO	BPA	10 ppm, Xe lamp as a simulated sun light source (500 W); dose 800 mg/L; time 3.5 h	80	(Peng Xu et al. 2021)
Zn-doped Cu_2_O	CIP	20 ppm, metallic halide lamp (400–1100 nm; 500 W); dose 600 mg/L; time 4 h	94.6	(Yu et al. 2019)
Ag-ZnO	Atenolol	5 mg/L, Tungsten halogen lamp (300 W), pH 8.5, 1 g/L catalyst dose, time 2 h	70.2	(Ramasamy et al. 2021)
ZnO/SnS_2_	CIP	10 mg/L, halogen lamp (> 400 nm; 200 W), pH 6.1, and catalyst dosage 0.5 g/L	-	(Makama et al. 2020)
ZnO/γ-Fe_2_O_3_	Tetracycline	30 mg/L, halogen lamp, pH 6.7, catalyst dose 15 mg/L, 150 min	88.52	(Semeraro et al. 2020)
gC_3_N_4_/NiO/ZnO/Fe_3_O_4_ nano-heterostructures	Esomeprazole	Visible light irradiation within 70 min	95.05 ± 1.72%	(Raha and Ahmaruzzaman 2020)
ZnO NPs	Tetracycline and ibuprofen	< 5 ppm, catalyst doses 10 and 0.5 mg/L, pH value from 7 to 9	Approx. 90%	(Choina et al. 2015)
ZnO NPs	MB MO	180-min sunlight irradiation	75% 87%	(Ghaffar et al. 2023)
ZnO NPs	Triclosan (TCS) and imidacloprid (IMD)	30 min under UV light	99.60% 96.09%	(Kaur et al. 2023)
W/Ag/ZnO nanocomposite	Turquoise Blue Dye (TBD)	170–200 mg/L, adsorbent dose of 0.1–0.01 g, pH range 2–3, contact time 60 min, 35 °C, under visible light exposure	Maximum absorption	(Noreen et al. 2022)
ZnO NPs, ZnO-rGO-flowers, and ZnO-rGO-rods	Polychlorinated biphenyls (PCBs)	10 µg/mL, UV lamp (365 nm, 100 W), catalyst dose 0.16 g/L, 8 h	74.1%, 92.4%, and 95.6%	(Merlano et al. 2022)
CdO-ZnO nanocomposites	Rhodamine B (RhB) dye	20 ppm, 120 min of UV light exposure (125 W mercury lamp), catalyst dose 0.5 g/L	87%	(Umar et al. 2022)
ZnO/NiFe_2_O_4_ nanocomposites	Rhodamine B MB	3 h under natural sunlight	98% 97%	(Stiadi et al. 2023)
ZnO/boron nitride quantum dots (BNQDs) BNQD_*x*_ (*x* = 1, 2, 4, and 6 wt.%)	MB MO	UV light irradiation	90.6–97.9%	(D. Liu et al. 2022)

The studies presented in Table 1 show the recent interest in using ZnO-based nanomaterials to degrade some of the most common emerging pollutants found in wastewater, like tetracycline (Patehkhor et al. 2021) (Xiao Chen et al. 2020b), BPA (Peng Xu et al. 2021) (Bilgin Simsek et al. 2018), MB (Lin et al. 2018), and metformin (Khavar et al. 2019). The studies present different methods used to enhance the ZnO NP photocatalytic performances by developing composites with other metal oxides and determining the optimal degradation conditions for the emergent pollutants studied.

Due to their large band gap energy (Eg > 3 eV), rapid recombination, and low charge-transfer rates of photoinduced electron–hole pairs, TiO_2_ and ZnO catalysts are partially limited (Jiang et al. 2014). It has been possible to create a wide range of hybrid catalysts to improve ZnO’s photoactivity and antiphotocorrosion. Studies showed that by using matched band energies, heterojunction between semiconductors can effectively separate photoinduced charge carriers and increase solar light absorption in the visible region (Ningning Wang et al. 2016). A study reported that rGO-based TiO_2_-ZnO nanostructures (rGO/TiO_2_/ZnO) were synthesized using a hydrothermal method and characterized through SEM, XRD, and XPS analyses that confirmed the formation of wurtzite ZnO and anatase TiO_2_ in the tandem nanostructure (Bilgin Simsek et al. 2018). The UV–Vis spectrum indicated that this hybrid catalyst possesses the lowest band gap energy (Eg = 2.5 eV). Photocatalytic degradation of bisphenol A, ibuprofen, and flurbiprofen was examined under UV and visible light irradiation. ZnO, TiO_2_, TiO_2_/ZnO, and rGO/TiO_2_ composites were prepared for comparison, with the rGO/TiO_2_/ZnO catalyst demonstrating superior photocatalytic performance under visible light irradiation. The enhanced degradation efficiency of the TiO_2_/ZnO structure by rGO is attributed to graphene’s electron properties, its role as a supportive substrate providing a two-dimensional structure, and the reduction of the band gap energy.

The successful chemical precipitation process was employed to synthesize spherical ZnO NPs of varying sizes using different solvents, namely, water (referred to as ZnOw) and ethanol (referred to as ZnOe) (Choina et al. 2015). In ethanol, the nanoparticles exhibited sizes ranging from 10 to 30 nm, while in the aqueous solution, they measured approximately 100 nm. During the photocatalytic decomposition experiments of two model drugs, tetracycline (TC) and ibuprofen (IBP), at two different photocatalyst concentrations (10 mg/L and 0.5 mg/L), distinct adsorption behavior was observed due to variations in specific surface areas and substrate concentrations. Notably, the adsorption of TC and IBP onto ZnOe and under low irradiation power was found to be greater than that onto ZnOw, particularly at lower photocatalyst-to-substrate mass ratios, which became apparent even below 10 ppm. Increasing the pH level from 7 to 9 resulted in a boost in the photocatalytic degradation of TC, from approximately 65 to 90% when using ZnOe and from around 50 to 85% when using ZnOw.

**Fig. 12:**
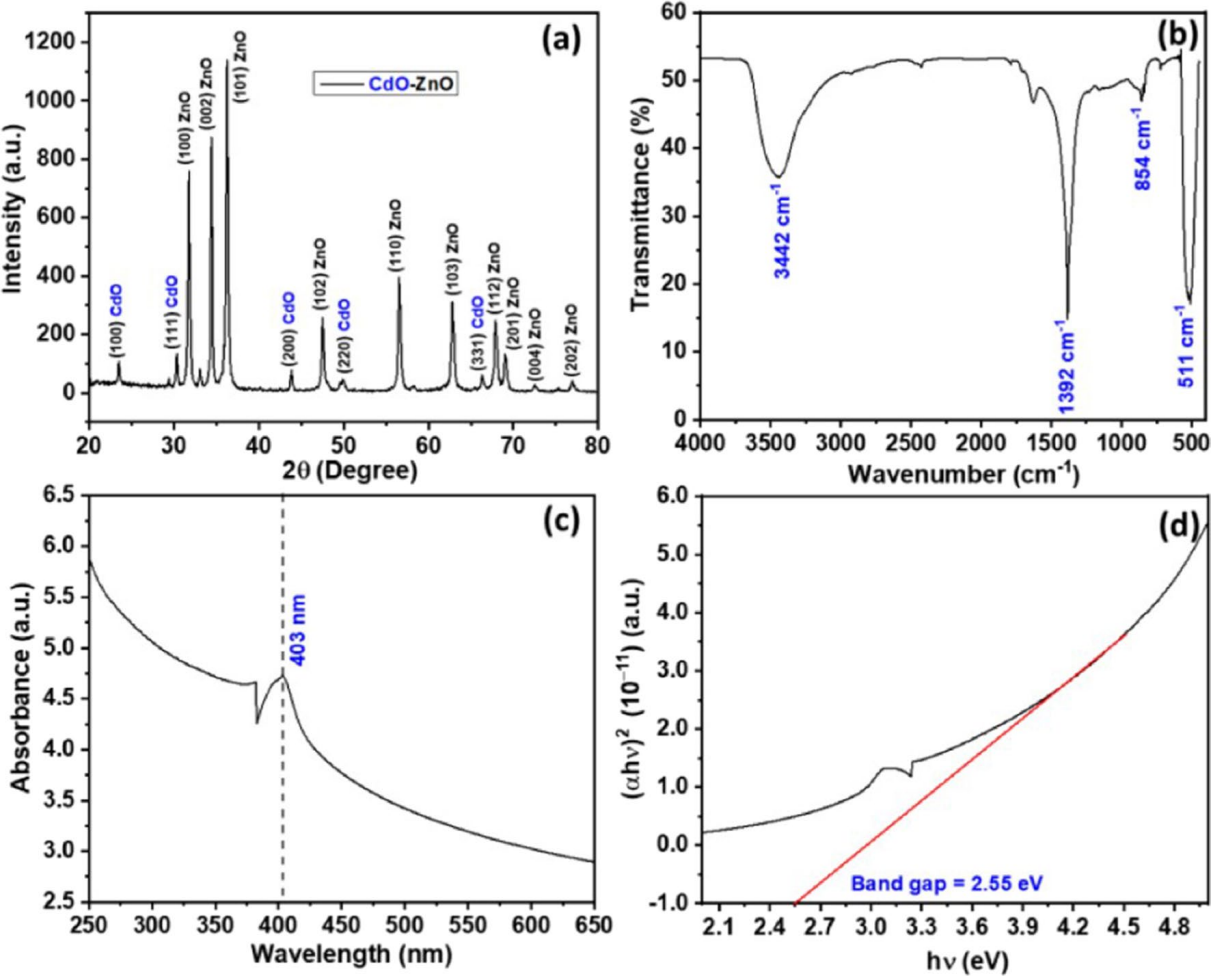
**a** XRD spectrum, **b** FTIR spectrum, **c** UV–Vis spectrum, and **d** Tauc’s plot for the evaluation the optical band gaps of the CdO-ZnO nanocomposites. The red line signifies the analysis of the linear region to determine the band gap at the *x*-axis intercept, while the black line depicts the changes in (αhν).^2^ versus hν (Umar et al. 2022) (open access)

CdO-ZnO nanocomposites were synthesized using a simple solution method for degradation of RhB dye (Umar et al. 2022). The characterization of morphological, structural, phase, vibrational, optical, and compositional properties of CdO-ZnO nanocomposites denoted the aggregates ranging from 250 to 500 nm in size formed after annealing at 500 °C and hexagonal wurtzite and cubic phases in ZnO and CdO, respectively, with a crystal size of 28.06 nm (Fig. 12a). The stretching vibration of the Zn–O and Cd–O bonds was evident from the prominent wide peak at 511 cm^−1^ (Fig. 12b). At room temperature, the primary absorption peak for the nanocomposites was observed at approximately 403 nm (Fig. 12c). The nanocomposites had a band gap energy of 2.55 eV (Fig. 12d), which was considerably smaller compared to pure ZnO nanostructures but higher than that of CdO nanomaterials (2.2–2.5 eV).

**Fig. 13:**
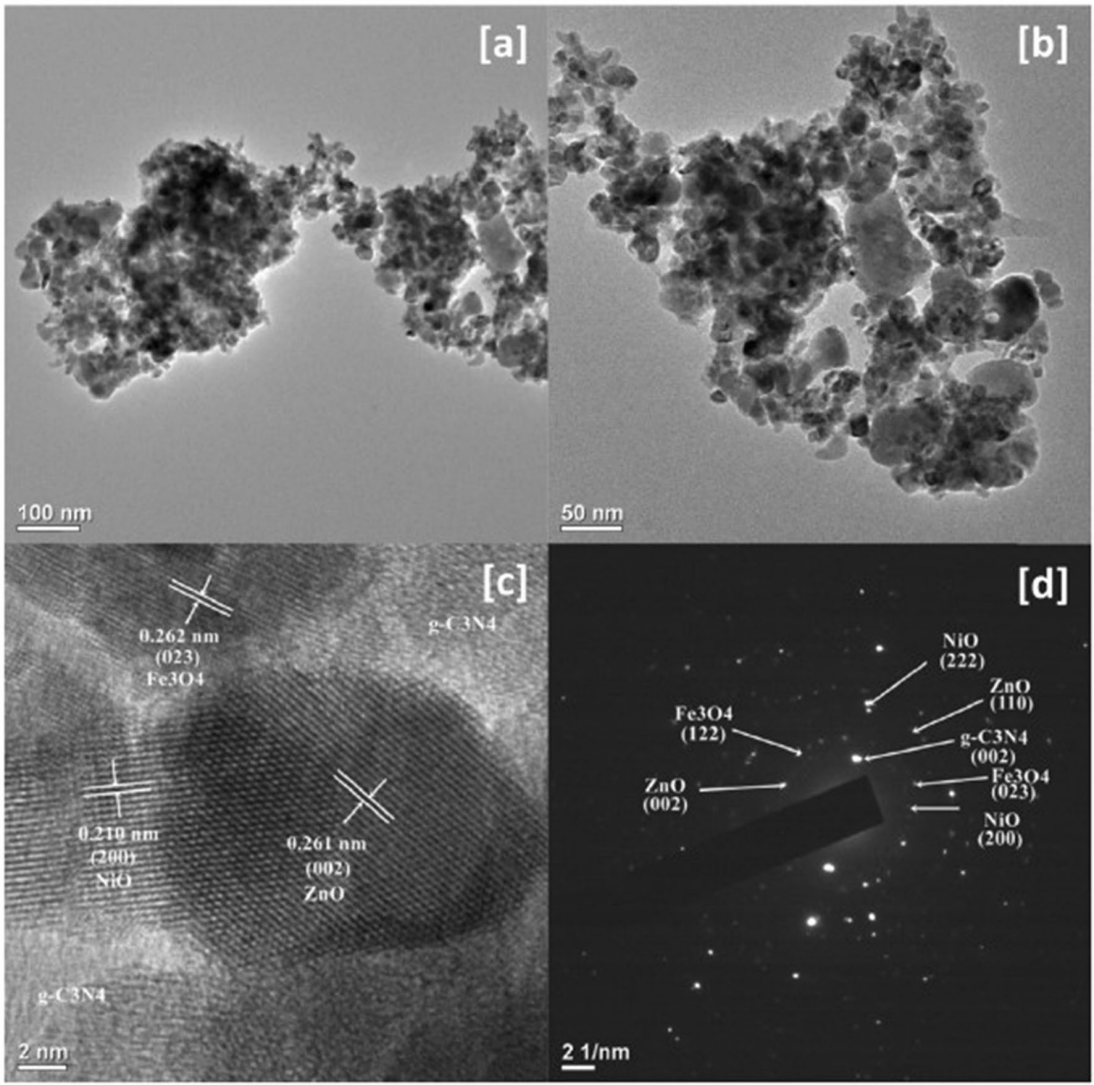
TEM (**a**, **b**) micrographs, HRTEM (**c**) micrograph, and SAED patterns (**d**) of gC_3_N_4_/NiO/ZnO/Fe_3_O_4_ (reused with permission from Raha and Ahmaruzzaman 2020)

Novel nano-heterostructures consisting of gC_3_N_4_, NiO, ZnO, and Fe_3_O_4_ were synthesized using a hydrothermal method at 110 °C for 18 h (Raha and Ahmaruzzaman 2020). These structures demonstrated remarkable photocatalytic activity, achieving the degradation of esomeprazole, an emerging organic water pollutant and model drug, to a level of 95.05% ± 1.72% under visible light irradiation within 70 min. The investigated reaction mechanism also indicated a pseudo-first-order kinetics due to the coupling of ∙O_2_ and ∙OH between NiO and ZnO which has a broad band and gC_3_N_4_ which has a narrow band gap. To characterize the morphology, size, and crystallography of the gC_3_N_4_/NiO/ZnO/Fe_3_O_4_ nano-heterostructures, transmission electron microscopy (TEM), high-resolution transmission electron microscopy (HRTEM), and selected area electron diffraction (SAED) were performed. The TEM images (Fig. 13a and b) revealed dispersed nanoparticles of ZnO, NiO, and Fe_3_O_4_ distributed across the gC_3_N_4_ sheet. The HRTEM image (Fig. 13c) allowed for the measurement of interplanar spacings and differentiation of lattice fringes, facilitating the identification of ZnO, NiO, and Fe_3_O_4_ NPs. The average particle size was determined to be 17.06 nm. The SAED pattern (Fig. 13d) displayed concentric rings, indicating the polycrystalline nature of the nanohybrid structure. Within the SAED pattern, distinct planes of ZnO ((0 0 2), (1 1 0)), NiO ((2 0 0), (2 2 2)), Fe_3_O_4_ ((0 2 3), (1 2 2)), and gC_3_N_4_ ((0 0 2)) were identified and marked.

Many studies have reported the use of precious metals like silver (Ag), gold (Au), and palladium (Pd) (Vaya and Surolia 2020) to improve band gap energy in ZnO nanocomposites for the degradation of various pharmaceutical products. Ag has become more significant among these metals due to its great solar light absorption and ability to suppress electron–hole recombination through surface Plasmon resonance (Kaur et al. 2018). The hydrothermal method can be used to obtain Ag_2_O/ZnO/rGO heterojunction photocatalysts used for the photocatalytic degradation of some BPA pollutants under simulated sunlight. Doping with Ag led to the lowering of the band gap of ZnO, which led to about 80% removal efficiency of BPA using 5% Ag and 3% GO by weight. The reuse of Ag_2_O/ZnO/rGO is possible, the material showing good photostability and pH adaptability (Peng Xu et al. 2021). It was reported that despite having a small band gap energy (2.73 eV), the silver iodide (AgI) photosensitive semiconductor significantly increases photodegradation activity in composite PGCN/AgI/ZnO/CQDs due to strong contacts that promote photon transport and prevent rapid electron–hole pair recombination (Hasija et al. 2019).

**Fig. 14:**
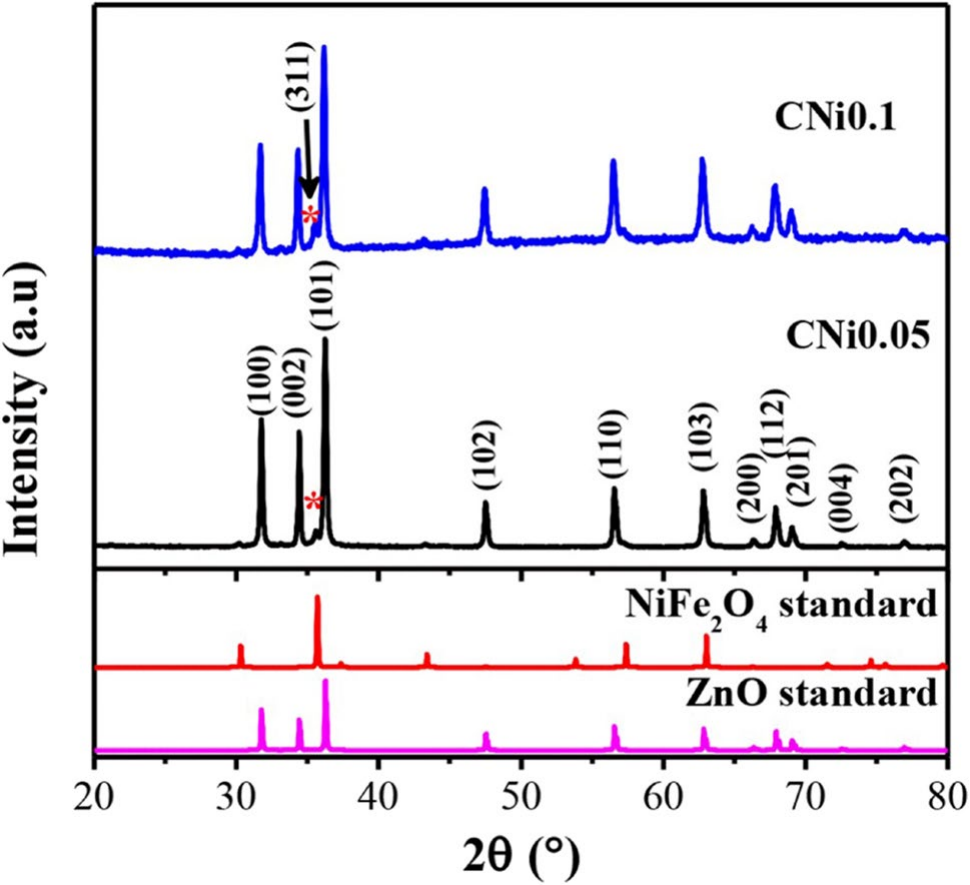
The XRD pattern for CNi0.05 and CNi0.1 nanocomposites (Stiadi et al. 2023) (open access)

ZnO/NiFe_2_O_4_ nanocomposites were synthesized through the hydrothermal method, with varying mole ratios of Zn^2+^ to NiFe_2_O_4_ (1:0.05 and 1:0.1), denoted as CNi0.05 and CNi0.1 (Stiadi et al. 2023). These nanocomposites were tested for their ability to degrade Rhodamine B and methylene blue (MB) under natural sunlight. In both nanocomposites, the primary diffraction peaks were observed at 2*θ* values of 31.7°, 34.4°, and 36.2°, corresponding to the miller indices of (100), (002), and (101), which are indicative of the presence of ZnO NPs within the composites (Fig. 14). Additionally, both ZnO/NiFe_2_O_4_ nanocomposites exhibited specific NiFe_2_O_4_ peaks at 2*θ* = 35.7° with a 311 miller index, denoted by an asterisk (*), confirming the presence of this phase in the composite materials. The absence of any other XRD peaks, aside from those of ZnO and NiFe_2_O_4_, demonstrated the successful formation of single-phase ZnO/NiFe_2_O_4_ nanocomposites using this synthesis method.

In the absence of a catalyst, the degradation of Rhodamine B only reached 10%, while the degradation of MB reached 30% after 3 h. Notably, the CNi0.05 nanocomposite showed the highest degradation percentages for both dyes, when the degradation percentage reached 98% for Rhodamine B and 97% for MB at using the CNi0.05 composite as the catalyst. Without exposure to light, the degradation percentage of the CNi0.05 composite remained low at 6–8% after 3 h. These findings provide clear evidence that the degradation of dyes by CNi0.05 occurred through photocatalysis, as the irradiation process substantially enhanced the degradation percentages.

In another study Khavar et al. (2019), a novel nanostructured catalyst Fe_3_O_4_@rGO@ZnO/Ag NPs (FGZAg) is synthesized by using graphene oxide and ZnO-coated Fe_3_O_4_ microspheres doped with Ag NPs, and its efficiency in metabolizing metformin (MTF) is examined both in the ultraviolet and visible spectra of light. The resulting samples were analyzed using a variety of analytical techniques, which revealed onion-shaped spheres with a hexagonal wurtzite crystal structure and a mesoporous texture, as well as Ag NPs that were securely stuck to the catalyst’s surface. When degrading MTF under visible light, FGZAg showed significantly improved photocatalytic activity when compared to pure ZnO, completing total degradation and 60% mineralization of 20 mg/L MTF in just 60 min.

A nanomaterial made of ZnO and maghemite was used in a study by Semeraro et al. to speed up the photodegradation of tetracycline in aqueous solutions when subjected to sunlight. Iron oxide nanoparticles significantly altered the morphology of ZnO while keeping both iron oxide (γ-Fe_2_O_3_) and ZnO (wurtzite form) in their crystalline phases (Semeraro et al. 2020). The efficient removal of the photocatalyst from water using a weak external magnetic field was made possible by the paramagnetic properties of γ-Fe_2_O_3_ nanoparticles. This resulted in a significant reduction in separation time and suggested the use of the ZnO/γ-Fe_2_O_3_ nanocomposite for large-scale, continuous water treatment processes. Additionally, compared to bare ZnO, γ-Fe_2_O_3_ improved the nanostructures’ porosity and adsorption qualities, which is essential because adsorption is the first stage of photodegradation. The presence of γ-Fe_2_O_3_ enhanced catalytic activity by around 20% while having no negative effects on ZnO-mediated photodegradation. In conclusion, the ZnO/γ-Fe_2_O_3_ nanocomposite showed outstanding recyclability thanks to the paramagnetic qualities of γ-Fe_2_O_3_ and the ability to breakdown over 88% of water-dissolved tetracycline.

In order to successfully remove the difficult antibiotic ciprofloxacin (CIP) from wastewater, a microwave-prepared porous ZnO/SnS_2_ photocatalyst was used in visible light photocatalytic degradation (Makama et al. 2020). The research looked into the effects of radical scavengers on the degradation process as well as a number of process factors, including pH, catalyst dose, and initial CIP concentration. The findings showed that a pH of 6.1 and a catalyst dose of 500 mg/L were the ideal conditions for CIP degradation, while an excessive catalyst dosage hindered the reaction rate due to light scattering and decreased light penetration. In a different investigation, solvothermal preparation of Zn-doped Cu_2_O particles produced improved specific surface areas, increased visible light absorption, and a larger band gap than pure Cu_2_O (Yu et al. 2019). R_2_-Cu_2_O demonstrated the best photocatalytic performance and reusability, achieving a remarkable 94.6% degradation of ciprofloxacin. Even after five cycles, the degradation percentage remained above 91%.

To enhance even more the stability and photodegradation properties of ZnO NPs, studies have reported the preparation of novel matrices with eco-friendly supports like clays and carbon-based materials that are further discussed in detail in the next chapter.

## New reliable photocatalytic matrix based on ZnO nanostructures

The efficiency of a photocatalyst is given by its stability and the possibility of rapid recovery. Thus, the integration of ZnO NPs, which offer increased reactivity and catalytic potential due to their reduced dimensions, can also lead to significant losses in the analyzed systems. To address this issue, the creation of appropriate matrices has been studied in recent times. Below, we present the most efficient variants of using certain supports for ZnO NPs in the degradation process of EPs. In order to improve the growth surface of ZnO nanostructures on the support materials, carbon-based substrates can be used: activated carbon (AC), graphene oxide (GO), carbon nitride (C_3_N_4_), and graphene (Le et al. 2022). Another type of support is clay, which was used for the in situ assembly of ZnO nanoparticles to obtain nanoarchitectures. Obtaining them consisted in the incorporation of the metal precursor of Zn acetylacetonate in suspensions different from clays, the product obtained being calcined (Akkari et al. 2016). Table 2 describes the performances of such ZnO catalyst with different supports.

**Table 2:** Photocatalytic degradation efficiencies of ZnO-based composites for EPs present in water and wastewater

Support	Catalyst	Preparation method and material morphology	Pollutants, degradation conditions	Ref
AC	ZnO	Hydrothermal, nanorods	MB, 5 ppm, 120 min, UVA, 77% PE	(M. Malakootian et al. 2020)
		Sonochemical, nanoparticles	Reactive red, 200 ppm, 720 min, UVC, 87% PE	(Aber et al. 2019)
		Precipitation, nanoparticles	Congo red, 30 ppm, 120 min, solar light, 100% PE	(Raizada et al. 2014)
		Hydrothermal, nanoflower	Amoxicillin, 10 ppm, 65 min, UVA, 95% PE	(Guihua Chen et al. 2014)
	ZnO/TiO_2_	Sonochemical, nanoparticles	Black treacle, 5 ppm, 60 min, UVC, 96% PE	(Benton et al. 2016)
	ZnO/ZnS	Impregnation, nanoparticles	Reactive blue, 20 ppm, 90 min, visible, 33% PE	(Ma et al. 2011)
GO	ZnO	Hydrothermal, nanorods	MB, 10 ppm, 120 min, solar, 94% PE	(Ranjith et al. 2017)
		Hydrothermal, nanorods	MB, 5 ppm, 130 min, UVA, 88% PE	(Xun Zhou et al. 2012)
		Precipitation, dumbbell	MB, 10 ppm, 180 min, UV–Vis, 95% PE	(Prabhu et al. 2019)
		Hydrothermal, nanoparticles	MO, 5ppm, 30 min, UVA, 95% PE	(Tayyebi et al. 2016)
		Hydrothermal, nanoflower	Ofloxacin, 20 ppm, 300 min, UV, 99% PE	(Pushkal Sharma et al. 2020)
	ZnO/Ni	Sonochemical, nanoparticles	Brilliant green, 20 ppm, 90 min, visible, 100% PE	(Peter et al. 2019)
	ZnO/Ag_2_O	Hydrothermal, nanoflower	BPA, 10 ppm, 180 min, solar, 80% PE	(Peng Xu et al. 2021)
	Eu^3+^/ZnO/Bi_2_O_3_	Precipitation, nanorods	Dimethyl phenol, 10 ppm, 120 min, UVB, 99% PE	(Shandilya et al. 2020)
C_3_N_4_	ZnO	Precipitation, nanoparticles	MB, 10 ppm, 120 min, visible, 60% PE	(Xu Tan et al. 2019b)
	AgI/ZnO	Precipitation, nanoparticles	Dinitrophenol, 10 ppm, 120, visible, 98% PE	(Hasija et al. 2019)
Graphene	ZnO	Hydrothermal, nanoparticles	Deoxynivalenol, 15 ppm, 30 min, UVC, 99% PE	(Xiaojuan Bai et al. 2017)
	ZnFe_2_O_4_/ZnO	Sonochemical, nanoparticles	MB, 10 ppm, 120 min, solar, 98% PE	(Sun et al. 2013)
C-dots	ZnO	Spin-coating process, nanorods	MB, 10 mM, 70 min, UV lamp (20V), 74.98% PE	(Roza et al. 2020)
		Solvothermal, nanospheres	MB, 3.1 × 10^−5^ M, 30 min, UV halogen lamp (500 W), 96% PE, solar light 97% PE	(Velumani et al. 2020)
CDs/gC_3_N_4_	ZnO	Impregnation-thermal, nanorods	Tetracycline, 2.3 × 10^−5^ M, 30 min, visible xenon lamp, 100 PE%	(Guo et al. 2017)
CQDs	ZnO@HNTs	Precipitation, nanospheres	Tetracycline, 4.5 × 10^−5^ M, 90 min, xenon lamp (500 W), 92.48 PE%	(Jinze Li et al. 2019)
GQD	ZnO	Hydrothermal, nanorods	MB, 1 × 10^−5^ M, 70 min, natural sunlight, 95% PE Carbendazim, 1 × 10^−5^ M, 70 min, natural sunlight, 94% PE	(Suneel Kumar et al. 2018b)
N-CQDs	Ni-ZnO	Hydrothermal, nanospheres	MB, 1 × 10^−5^ M, 120 min, visible light (400 W), 87% PE	(Behnood and Sodeifian 2020)
NPCQD	ZnO	Hydrothermal, nanospheres	MB, 3.1 × 10^−5^ M, 30 min, daylight xenon lamp (300 W), 90% PE	(Song et al. 2019)
N-GQDs	ZnO	Hydrothermal, nanospheres and nanoplates	MB, 9.4 × 10^−5^ M, 120 min, mercury lamp (250 W), 100% PE	(Sodeifian and Behnood 2020)
CQD/N	ZnO	Precipitation and mixing, nanospheres	Malachite green, 1 × 10^−4^ M, 30 min, natural daylight, 100% PE MB, 1 × 10^−4^ M, 45 min, natural daylight, 100% PE Fluorescein, 1 × 10^−4^ M, 15 min, natural daylight, 95% PE	(Muthulingam et al. 2015)
Clay	ZnO	Mixing precipitation, nanoparticles	IBP, 10 ppm, 600 min, UVB, 73% PE	(Akkari et al. 2018a)
		Impregnation, nanoparticles	MB, 3 ppm, 120 min, UVB, 98% PE	(Akkari et al. 2016)
		Hydrothermal, nanoparticles	Levofloxacin, 30 ppm, 75 min, visible, 100% PE	(Abukhadra et al. 2020)
	ZnO/TiO_2_	Sol–gel, nanoparticles	Methylene green, 75 ppm, 30 min, UVA, 100% PE	(Bel Hadjltaief et al. 2016)
Expanded clay	ZnO	Precipitation, nanoparticles	Reactive yellow, 50 ppm, 45 min, UVC, 99% PE	(Moradi et al. 2015)
Tunisian clay	ZnO	Sol–gel, nanoparticles	Congo red, 50 ppm, 120 min, UVA, 100% PE	(Hadjltaief et al. 2018)
Clinoptilolite	ZnO	Sonoprecipitation, nanorods	Furosemide, 15 ppm, 90 min, UVA, 80% PE	(Heidari et al. 2020)
Halloysite	ZnO	Precipitation, nanoparticles	MB, 10 ppm, 90 min, UVA, 99% PE	(Peng et al. 2017)
		Precipitation, nanoparticles	Rhodamine B, 5 ppm, 20 min, UVB, 88% PE	(Massaro et al. 2020)
	N/ZnO	Impregnation, nanoparticles	MO, 20 ppm, 480 min, Solar, 95% PE	(Cheng and Sun 2015)
Montmorillonite	ZnO	Thermal attachment, nanoparticles	Metronidazole, 25 ppm, 30 min, UVA, 100% PE	(Khataee et al. 2017)
		Precipitation, nanoparticles	Disperse red, 100 ppm, 5 min, UVC, 82% PE	(Kıranşan et al. 2015)
		Impregnation, nanoparticles	MB, 10 ppm, 60 min, UVB, 92% PE	(Fatimah et al. 2011)
Zeolite	ZnO/Fe_2_O_3_/MnO_2_	Hydrothermal, nanoparticles	MB, 10 ppm, 120 min, Visible, 93% PE	(Tedla et al. 2015)
	ZnO/Cu	Precipitation, nanoparticles	MO, 9 ppm, 120 min, UVC, 90% PE	(Karimi Shamsabadi and Behpour 2021)
Polyaniline (PANI)	ZnO	In situ chemical polymerization process, nanoparticles	MB, 50 ppm, 120 min, 90% PE	(Qin et al. 2018)
	ZnO	Arc-discharge method submerged in de-ionized water for ZnO preparation, and chemical polymerization process for the composite, a core–shell structure	MB, MG, catalyst concentration: 0.4 mg/mL; initial concentration of dyes, 1 × 10^−5^ M, 5 h, 99% PE for both	(Eskizeybek et al. 2012)
	ZnO	Precipitation for ZnO NPs, chemical polymerization process for the composite	MO, MB, 98.3% for MO and 99.2% for MB	(Saravanan et al. 2016)
	ZnO	In situ chemical polymerization	Metronidazole (MNZ), 10 ppm, catalyst dose = 1.0 g L^−1^; pH 7	(Asgari et al. 2019)
Polypyrrole (PPy)	ZnO	In situ chemical polymerization	DCF, 10 ppm, catalyst dose 1 g/L, xenon lamp, 60 min, 81% PE	(Silvestri et al. 2019)
Chitosan	TiO_2_–ZnO	Ultrasound assisted sol–gel method, nanoparticles	Tetracycline, 20 ppm, catalyst dose 0.5 g/L, pH 4, room temperature, 3 h 97.2%	(Patehkhor et al. 2021)
	ZnO NPs	Sol–gel synthesis	MB (6 × 10^−5^ M), catalyst dose 30 mg, 60 min, 60%PE	(Ben Amor et al. 2022)

### ZnO NPs—carbon-based supports

This section discusses the utilization of ZnO composites with carbon-based supports for the removal of different emerging pollutants from wastewater. In order to demonstrate the efficiency of photocatalytic degradation, laboratory tests are performed by varying some working parameters, starting from the time interval, the pH value, catalyst quantities, pollutant concentrations, etc. (Pushkal Sharma et al. 2020). For example, Sharma et al. indicated in the case of the reduced graphene oxide (rGO)-ZnO composite different contents of graphene, time, variable pH between 5 and 9, and different intensities of the UV lamp, in order to study the photocatalytic effect on the degradation of 20 mg/dm^3^ of ofloxacin (Pushkal Sharma et al. 2020). The efficiency was demonstrated by the degradation of about 99% of this pollutant, after about 6 h using 0.5% rGO-ZnO composite. Regarding the reuse process of the nanophotocatalyst, it was recovered by centrifugation, keeping the degradation efficiency after the first cycle from 99 to 96.4% and reaching an efficiency of about 83.3% after 6 cycles.

Photocatalytic composites with ZnO have the advantage of increasing the degradation efficiency of emerging pollutants. For example, in the case of BPA, photocatalytic tests using 5%-Ag_2_O/ZnO lead to about 87% degradation, after 180 min. The addition of 3% by weight of rGO led to the increase of the specific surface area and leads to an efficiency of about 80% in terms of BPA degradation, and the recombination rate is lower for the photoinduced electron–hole pairs of 5%-Ag_2_O/ZnO/rGO-3% (Peng Xu et al. 2021).

Mesoporous zinc oxide can be deposited on a reduced graphene support (rGO@ZnO), with efficiency in the photocatalytic mineralization of ofloxacin from aqueous solution. The literature indicates the obtaining of nanophotocatalysts in situ with different contents of GO (0.2%, 0.5%, 1%, and 2%) using zinc nitrate, with pH control of about 9, in the presence of ammonia liquid at 110 °C for 7 h (Pushkal Sharma et al. 2020).

Graphene oxide (GO)/ZnO composite was used to degrade atenolol, a beta-blocker used to regulate blood pressure, by artificial irradiation, achieving a degradation efficiency of 85% at a catalyst dosage of 1.2 g/L, pH 4 after 60 min (Bhatia et al. 2021). They also observed that GO-ZnO outperformed GO-TiO_2_ due to a faster reaction rate, highlighting its superior photocatalytic performance.

ZnO/CdO/rGO was obtained by the hydrothermal method in order to test for the photocatalytic degradation of BPA, ThB, and CIP under UV light illumination (Sonu Kumar et al. 2022). The ZnO-CdO incorporated with reduced graphene oxide (ZCG)-5 nanocomposite can lead to the degradation and mineralization of BPA by 98.5%, thymol blue (ThB) by 98.38%, and CIP by 99.28% after UV light irradiation. ZCG-5, through its good photocatalytic activity, leads to the generation of more ROS species, especially as a result of the incorporation of rGO nanosheets with ZnO-CdO in the photocatalyst (Sonu Kumar et al. 2022).

The graphite powder was pre-oxidized using P_2_O_5_ and concentrated sulfuric acid, and the product obtained, after drying, was further oxidized in the presence of NaNO_3_, H_2_SO_4_, and KMnO_4_. After the complete reaction, hydrogen peroxide was added to finalize the process in order to obtain graphene oxide (GO). The synthesis of the ZnO/CdO/rGO and ZnO/CdO composites took place in an autoclave by using specific metal precursors of the type Zn(N0_3_)_2_·6H_2_0 and respectively Cd(N0_3_)_2_·4H_2_0, in the presence of GO, in a basic environment (Shouli Bai et al. 2020).

Hu et al. obtained ZnO NPs by the assisted wet chemical method, using zinc acetate in NaOH medium, which were further loaded into biochar by sonication (Hu et al. 2019). A carbon/ZnO heterojunction is formed that leads to high degradation efficiency for dyes, the advantages being the stability of these materials, the large pore volume, and the high adsorption capacity (Srikanth et al. 2017). These materials can be obtained by hydrothermal, sonochemical, precipitation, electrochemical, and immersion coating methods (Le et al. 2022).

ZnO can be doped with N doped with carbon sheets (N carbon@N-ZnO) obtained from bacterial cellulose biomass (BC) for the removal of persistent pharmaceutical substances such as tetracycline and di-chlorophenol. The degradation efficiency of tetracycline was about 97%, with visible light.

The literature also indicates photocatalysts obtained by green synthesis in the form of a quaternary nanocomposite of the P-doped graphitic carbon nitride (PGCN)/AgI/ZnO/CQD type, from bamboo leaves, used for photocatalysis assisted adsorption at pH 4 of 2,4-dinitrophenol, with an efficiency of about 98% in 2 h (Hasija et al. 2019). The efficiency of approx. 89% after 10 reuse cycles was also worth noting. According to the known reaction mechanisms, CQD completes the degradation rate, the carbon source leading to an increase in the adsorption rate and a reduction in the recombination rate (Hasija et al. 2019).

ZnO NPs show photocatalytic performance under UV light in the treatment of pharmaceutical pollutants such as cloxacillin and ciprofloxacin (CIP), and triangular silver nanoplates (T-Ag)/ZnO and ZnO/N,S-doped carbon quantum dots (N,S-CQDs) nanoflowers showed high efficiency for the degradation of norfloxacin in light visible, due to the synergistic and surface plasmon resonance effect of T-Ag on ZnO nanoflowers as well as the transfer of photogenerated electrons from the ZnO conduction band to the N,S-CQD surface (Verma et al. 2021; Elmolla and Chaudhuri 2010; Shi-Lin Zhou et al. 2016; Yanning Qu et al. 2020).

The photocatalytic activity of ZnO/N,S-CQD nanoflowers under simulated sunlight is about 92.9% and 85.8% of CIP respectively was degraded at 20 min and 50 min respectively. The degradation efficiency of cephalexin (CEL) was 86.7% after 50 min (Yanning Qu et al. 2020).

Qu et al. carried out tests on the photodegradation of organic pollutants in real water, using ZnO/N,S-CQD nanoflowers under simulated sunlight irradiation for CIP, CEL, and MB. The waters were fortified with pollutants where there was only one. When the fortification was made with CIP and MB, the degradation was about 73.6% and 95.1%, respectively. In the case of CIP and CEL, the degradation was approximately 71.7% and 70%, respectively. In the case of the CIP-CEL-MB triple system, the efficiency for antibiotics was about 60% and 94.9% for MB (Yanning Qu et al. 2020). The ZnO/N, S-CQD hybrid composite obtained by a hydrothermal method tested for CIP varied from 92.9% under simulated light at 20 min to about 85.8% for 50 min under natural light (Mei et al. 2022; Yanning Qu et al. 2020).

Although camphor leaf biochar was created as an adsorbent for CIP removal, its effectiveness was constrained by its low specific surface area and adsorption capacity. A novel technique for producing magnetic biochar enhanced with ZnO nanoparticles was created to address this. A dose of 0.2 g/L of the resultant ZnO/biochar nanoadsorbent was tested for the removal of CIP at pH 4 and 40 °C, obtaining a maximum adsorption capacity of 449.40 mg/L after 24-h contact time. The adsorption mechanism involved π-π interaction, H-bond, electrostatic interaction, and hydrophobic interaction.

ZnO/N,S-CQD nanoflowers were obtained by the separate synthesis of N,S-CQD by hydrothermal treatment of carbonized L-cysteine with nitric acid in the presence of ethylene glycol as a passivating agent. ZnO NPs were obtained from precursor Zn(NO_3_)_2_·6H_2_O, the obtained solutions being mixed in different proportions with N,S-CQD, then placed in an autoclave at 100 °C for 12 h (Yanning Qu et al. 2020).

Compared to other metal oxide semiconductor photocatalytic materials, ZnO exhibits better photosensitivity and photochemical stability (M Arunpandian et al. 2020). There are numerous studies on the photodegradation of organic compounds, mainly dyes such as MB, Rhodamine, benzoic acid, and Congo red (Al Ja’farawy et al. 2022). The advantage of using ZnO is that the degradation of pollutants can take place by using sunlight, through the active sites and hydroxyl radicals on the surface of the photocatalyst (Bhuyan et al. 2015). However, in order to ensure a good dispersion, stability, and biocompatibility with the environments in which these NPs are used, quantum dot (CQD) particles in combination with ZnO offer high electronic conductivity and tunable photoluminescence, and the functional groups on the CQD surface stimulate adsorption of dyes.

Carbon nanostructures show electron storage capacity and can be combined with ZnO NPs (Ru Wang et al. 2017a). CQDs can act as an electron sink by preventing the recombination of electron–hole pairs. It is believed that CQDs can act as mediator electrons to increase the efficiency of visible light separation, increasing the concentration of free radicals such as O_2_∙ and ∙OH (Al Ja’farawy et al. 2022; Ru Wang et al. 2017a). The photocatalytic degradation mechanism is based on photoexcited electron transfer from CB of ZnO to CQD. The presence of CQDs delays the recombination of charge carriers due to the carbon nanostructure that offers a high electron storage capacity (Al Ja’farawy et al. 2022).

The advantages of synthesized composite in comparison with simple ZnO are the narrowing band gap, decreasing recombination rate, surface roughness, and greater ability to absorb dye molecules. The synthesis of different ZnO-rGO and ZnO-GO nanocomposites has received a lot of attention. The development of ZnO-rGO and ZnO-GO heterostructures has been found to increase light absorption, enhance charge separation and transportation, and lengthen the functional lifetime of photocatalysts, according to studies that have been conducted to date.

Various methods for synthesizing ZnO-rGO and ZnO-GO nanocomposites involve incorporating ZnO NPs onto the surfaces of GO or rGO materials (Yaqoob et al. 2020b). ZnO NPs and nanorods offer advantages such as large surface areas, providing numerous active sites for pollutant adsorption and photodegradation, high ROS generation under UV light, chemical stability, and scalable synthesis. However, challenges like high photocorrosion activity and low photosensitivity under visible light hinder their potential use. In contrast, GO and rGO have been proposed as excellent ZnO substrates due to their high surface areas and active adsorption sites. The formation of ZnO-rGO and ZnO-GO nanocomposites shows promise for wastewater decontamination, but other morphologies and reactor integration have been underexplored.

Reduced recombination losses in ZnO-rGO or ZnO-GO heterostructures are the cause of the increased photocatalytic activity in ZnO-rGO and ZnO-GO nanocomposites. Further research on visible light photodegradation processes is necessary because of their problematic performance under visible light. Furthermore, there has not been much discussion in the literature about the recyclable and reusable nature of these nanocomposites. To fully realize the potential of photocatalysis for wastewater treatment, a comprehensive strategy that combines reactor design and photocatalyst production is required. The fundamental technical and scientific obstacles preventing their employment in technologically and industrially relevant applications must be overcome, although there are still substantial problems to be solved.

### ZnO NPs—clay-based supports

The immobilization of ZnO NPs for the controlled use of ZnO in photocatalytic processes can also take place on clay minerals such as montmorillonite, bentonite, halloysite, sepiolite, and zeolite (Le et al. 2022). The use of these minerals has the advantage of availability and low cost. Immobilization methods can be by precipitation, sol–gel, impregnation, and hydrothermal (Akkari et al. 2018b, 2016; Khataee et al. 2017; Fatimah et al. 2011; Hadjltaief et al. 2018).

ZnO NP/clay-type photocatalyst with dimensions between 9 and 13 nm shows high degradation and adsorption efficiencies for methylene green of about 90% and for Congo red of about 88%. Adsorption is dependent on the pH and functional groups of the dye. Based on UV, the dyes adsorbed on the ZnO/clay surface are mineralized by OH∙- and O_2_∙^−^-type reactive species. The immobilization of ZnO on clay led to the increase of active sites on the ZnO surface necessary for the formation of reactive oxygen species (Le et al. 2022).

The major disadvantage also appears in the separation process, which remains quite difficult, so that recovery is also challenging (Siahpoosh and Soleimani 2017; Massaro et al. 2020; Peng et al. 2017). The literature indicates obtaining a ZnO-Fe_3_O_4_ composite immobilized on sepiolite (Akkari et al. 2016). The advantage of such a composite is represented by the magnetic properties of Fe_3_O_4_, which can help to efficiently separate the material from water, after being used as a photocatalyst for the degradation of IBP under sunlight. The degradation was about 88%, the recovery and reuse of ZnO/Fe_3_O_4_-sepiolite being possible after several operating cycles.

Furosemide represents one of the major emerging pollutants and photocatalytic degradation is a viable option demonstrated at different concentrations with different power lamps (1000 W Xe, 125 W Hg, and 8 W UVA) (Heidari et al. 2020). Another photocatalyst based on ZnO deposited on clay with an intermediate carbon layer used in the degradation of estrogens (E1, E2, E3, EE2), included in the EPs class, led to the degradation of about 90% with normal illumination (Bayode et al. 2021b).

The reuse of the ZnO/ion exchange clinoptilolite nanophotocatalyst (ICLT) was possible for 5 consecutive cycles, showing excellent chemical stability. In this sense, it can be observed that after the 5th cycle, the nanophotocatalyst still shows 93.7% of its initial activity, due to the strong bonds created between the ZnO NPs and the ICLT support.

The nanophotocatalyst ZnO/ICLT prepared by sonoprecipitation demonstrated high photocatalytic activity for the degradation of furosemide. The zinc acetate precursor in the presence of NaOH was introduced into a homogeneous suspension dispersed by zeolite, which after about 2 h led to the formation of a precipitate. This by calcination led to homogeneous nanoarchitectures (Heidari et al. 2020).

An example of the efficiency of these supports is the degradation of CIP at pH 7, 30 min, up to about 96%, according to pseudo-prime and Langmuir–Hinshelwood kinetics. The nanophotocatalyst was obtained by synthesizing ZnO NPs using the thermal method and immobilizing them on the surface of granular porous stones. Zinc acetate was used to obtain thin films of ZnO that were deposited on the stones by the immersion coating method, followed by oven drying (Mahdizadeh et al. 2015; Mohammad Malakootian et al. 2019b).

IBP shows stability through UV irradiation, an efficient solution, due to the anionic form present in the solution being the direct adsorption on the clays. Nanoarchitectures of the ZnO/sepiolite type led to degradations of about 75% after 10 h, due to the presence of ZnO NPs (Akkari et al. 2018a).

ZnO NPs deposited on a stone support represent a photocatalyst with performance in the decontamination of waters contaminated with pharmaceutical pollutants and those originating from the textile industry. The elimination of phenazopyridine from wastewater can take place in a proportion of about 70% at a pH of 6.0, using this photocatalyst. Chemical oxygen demand (COD) values indicate a removal of more than 50% at natural pH (Mahdizadeh et al. 2015).

Pharmaceutical products detected in waters as EPs present increased risks for the safety of people and the environment. Photocatalytic techniques present a solution for ACE and ANT products, when the photocatalyst is a TiO_2_-ZnO/clay nanoarchitecture. The photocatalysts were obtained by the modified sol–gel method, Ti and Zn precursors were added to the clay dispersion, and the resulting gel, after drying, was calcined at 500 °C (Tobajas et al. 2017). TiO_2_-ZnO nanoparticles incorporated on the surface of a clay indicated the possibility of total degradation of ACE and ANT, after 10 h, under sunlight, due to the heterojunction that reduces electron–hole recombination. The photocatalyst showed stability after 4 operating cycles (Tobajas et al. 2017).

Low surface area, quick aggregation, and small particle size are some of ZnO NPs’ drawbacks, which make it difficult to recover them from aqueous solutions. Furthermore, releasing ZnO NPs into the environment can be hazardous to human health and the ecosystem. Immobilizing ZnO NPs onto a suitable matrix provides a remedy for these problems. In comparison to unsupported metal oxide nanoparticles, supported nanoparticles often have greater adsorption capacity, mechanical characteristics, thermal stability, and a higher specific surface area. Due to its low cost and wide availability, clay stands out as the best material to use for this. Clay’s natural structure also offers significant physical and chemical qualities such specific surface area, water retention capacity, ion exchange capability, and reactivity (Bel Hadjltaief et al. 2016).

The combination of ZnO nanoparticles with Tunisian clay (referred to as ZnO clay) effectively adsorbed and removed malachite green and Congo red dyes (Hadjltaief et al. 2018). When subjected to simulated solar light, the ZnO-clay composite exhibited superior photocatalytic performance in comparison to UV irradiation. The composite’s zero point charge (pHpzc) was found to be 6.58 in the neutral pH range, rendering it a highly efficient adsorbent and photocatalyst for both positively charged (cationic) and negatively charged (anionic) dyes. Consequently, the ZnO-clay composite could efficiently adsorb and photodegrade cationic dyes in alkaline conditions (pH > 8) and anionic dyes in acidic conditions (pH < 4) (Gusain et al. 2019).

### ZnO NPs—other supports

The literature also indicates different supports that can incorporate ZnO NPs from stainless steel wire (Abd Aziz et al. 2014; Linhua Xu et al. 2020) and mesh (Vu et al. 2013; Jung and Yong 2011; Xiaofei Wang et al. 2017b), ceramic plate (Aditya et al. 2019; Shavisi et al. 2016), stone (Mohammad Malakootian et al. 2019b; Mahdizadeh et al. 2015; Mohammad Malakootian et al. 2019a), shells (Shirzad-Siboni et al. 2014), aluminum (Al_2_O_3_) (Huihu Wang et al. 2012a; Stojadinović et al. 2020), Zn plate (Ramirez-Canon et al. 2018), Ni foam (Zhu et al. 2020), woven cotton (Baruah et al. 2019) and bamboo (Jin et al. 2014), and pineapple leaf fibers (Le et al. 2022; Deebansok et al. 2021).

A ternary Au-SnO_2_-CdS photocatalyst was used for the degradation of about 95% imidacloprid, at pH 4, according to a pseudo-first-order reaction, with a stability of about 15% after 6 cycles. The analyzed insecticide was also tested with other photocatalysts for which the removal rates were lower: tungstophosphoric acid HPW/TiO_2_ 83% and Ag-ZnO 52% (Mohanta and Ahmaruzzaman 2021).

ZnO with nanostar (NSt) morphology was also used in the composition of Ag@ZnONSt and Pd@ZnONSt photocatalysts for the degradation of methyl parathion (MPT), pendimethalin (PDM), and trifluralin (TFL). Pd@ZnONSt had 99.8% degradation efficiency and stability over six degradation cycles (Veerakumar et al. 2021).

A number of studies on nanostructured ZnO are related to their practical and secure integration into different macromolecular composites, such as polyesters (Yuan et al. 2016), polysaccharides, polyethylene glycol (Melinte et al. 2019), poly (N-isopropylacrylamide) (Podasca et al. 2016), and hybrid polymers (Nicolay et al. 2015). Based on the numerous reports referring to the enhancement of the photocatalytic efficiency of ZnO nanomaterials, polyaniline (PANI) has received significant interest as it has been considered one of the most promising materials for enhancing the electrochemical and photocatalytic performance of ZnO. The fact that methylene blue (MB) is usually often chosen as a model pollutant to assess the photocatalytic activities under UV/visible light irradiation at ambient temperature is another intriguing feature. According to Qin et al., PANI-ZnO NPs presented a high photocatalytic activity (reaction rate constant *k* = 1.944 × 10^2^ min^−1^) when removing a high concentration of MB (Qin et al. 2018).

By using an in situ chemical polymerization approach to create PANI-ZnO nanocomposites, one of the prerequisites for an effective photocatalyst has been met. Additionally, the glassy carbon electrode (GCE) was combined with PANI-ZnO hybrids created in this study, and the combined PANI-ZnO/GCE system was evaluated for its potential as a microbial fuel cell anode. Through the chemical oxidative polymerization of aniline, Eskizeybek et al. developed PANI/ZnO nanocomposites and looked into how MB and malachite green (MG) dyes in aqueous medium were degraded under both natural and UV light irradiation (Eskizeybek et al. 2012). The results showed that after 5 h of exposure to natural light, a dose of 0.4 g/L of PANI/ZnO nanocomposite photocatalyst degraded both dyes with 99% efficiency. PANI/ZnO nanocomposites with higher activity as a result of the intermolecular interactions and increased crystallinity between the conducting polymer and ZnO NPs were synthesized by Saravanan et al. They investigated the faster degradation of MB (*k* = 2.575 × 10^2^ min^−1^) in comparison with MO dye (*k* = 2.325 × 10^2^ min^−1^), which was likely due to the latter’s simpler structure (Saravanan et al. 2016).

The removal of metronidazole (MNZ), a synthetic antibacterial agent, from wastewater before it is released into the environment has drawn significant attention due to its non-biodegradability and high solubility in water. Asgari et al. recently looked into the ZnO/PANI nanocomposites’ photocatalytic capacity for degrading MNZ when exposed to UV and visible light. They found that the ZnO/PANI nanocomposite presented a rate of MNZ degradation (*k* = 2.53 × 10^2^ min^−1^) almost 63 times faster than that of the pure ZnO photocatalyst’s (*k* = 0.04 × 10^2^ min^−1^) (Asgari et al. 2019). In MNZ degradation, the importance of hydroxyl radicals (∙OH) and superoxide anion radicals (∙O_2_) was emphasized. Improved visible light absorption and a decrease in charge carrier recombination were related to the photocatalytic activity under UV and visible irradiation. After 6 cycles, the photocatalytic effectiveness under UV and visible irradiations only decreased by 9% and 8%, respectively. As a result, the findings demonstrated that the ZnO/PANI nanocomposite had excellent stability and could be utilized repeatedly.

The synthesis of composite photocatalysts using ZnO nanoparticles and polypyrrole (PPy) is also discussed in the literature. These composites were made using PPy to ZnO ratios of 5:1 and 25:1, respectively (Silvestri et al. 2019). By observing the breakdown of diclofenac (DCF) under artificial solar light, the photocatalytic activity of these PPy-ZnO composites was evaluated. The outcomes demonstrated that the PPy-ZnO 25:1 composite was approximately twice as efficient as pure ZnO and had a higher photocatalytic rate constant (*k* = 0.986 min^−1^) than the PPy-ZnO 5:1 composite. The sensitizing impact of PPy was said to be responsible for this improvement. However, it was demonstrated that a high PPy to ZnO ratio could result in flaws on the PPy surface that act as recombination centers for electron–hole pairs and lead to lower photocatalytic activity. The study also showed that after three consecutive cycles of degradation, the PPy-ZnO 25:1 composite maintained its photocatalytic activity, showing its potential for reuse.

Polysaccharides and biopolymers are commonly used as modifiers (capping agents) in the synthesis of biogenic ZnO NPs (Yaqoob et al. 2020a). Chitosan is a naturally occurring polysaccharide found in crustaceans and insects and has garnered considerable attention for its role in synthesizing metal and metal oxide NPs, including ZnO NPs. Chitosan possesses essential functional groups, including amino and hydroxyl groups, which play a crucial role in the removal of different pollutants from water. As a result, it is widely recognized as an environmentally friendly capping agent (size control agent) in the synthesis of various metal and metal oxide NPs (ZnO, MgO, TiO_2_) (Ben Amor et al. 2023). Previous research has demonstrated that chitosan offers several advantages when used for surface functionalization of metallic NPs, like the improvement of optical properties, enhancement of antimicrobial activity, and facilitation of drug loading and release (Ben Amor et al. 2022).

By using sol–gel and ultrasound-assisted techniques, several nanocomposites based on metal oxides and chitosan (TiO_2_-ZnO, TiO_2_-ZnO/CS, and TiO_2_-ZnO/CS-Gr) were developed (Patehkhor et al. 2021). These materials were then used under UV light to assess the photocatalytic degradation of tetracycline. The following BET, FESEM, EDX, FT-IR, and XRD techniques were used to characterize the produced materials. At the optimal operational conditions (tetracycline concentration of 20 mg/L, pH = 4, catalyst dosage of 0.5 g/L, and 3 h of irradiation time), the TiO_2_-ZnO with the 1:1 molar ratio supported with 1:2 weight ratio CS-Gr (T_1_Z_1_/CS_1_Gr_2_ sample) proved to be the most efficient composite achieving 97.2% photodegradation of tetracycline. As anticipated, chitosan and graphene significantly improved the results of the degrading process. Taking into account the used operational settings, this innovative photocatalyst is capable of treating pharmaceutical wastewater.

## Future trends and conclusions

It has been proven that ZnO NPs are an extremely versatile material with photodegradation activity, comparable or even better than chemically produced ones. Currently, the ZnO NP market includes the cosmetic industry, food products, coatings, sun care, paints, construction, buildings, antibacterial, and applications from materials science, optics, biomedicine, or electronics. This market is estimated to grow by 7.5% from 2022, so it will reach US$ 525.32 million by 2029.

The present research highlights the latest ecological strategies regarding the development of ZnO nanoparticles through simple, low-cost, and large-scale methods. The review provides a fundamental overview of green syntheses for ZnO NPs as a single material or embedded into a matrix compared to classical syntheses. The stability and minimization of losses of ZnO NPs in the environment after their use inspire recent research towards the identification of a stabilization matrix for these NPs. The main performances of ZnO NPs integrated into a stable, environmentally friendly matrix useful for EP degradation were presented, emphasizing the ZnO NP preparation method and material morphology linked with each EP degradation condition. ZnO NPs embedded into a carbon matrix support represent a viable alternative to current techniques and could also be obtained from waste materials.

This literature research was based on an extensive data collection from 2000 to present and selection method of the main articles cited in this review is described in the paper. The motivation of this study also lies in the current market situation regarding the performance of nanotechnologies in water decontamination. Considering the potential of ZnO NPs, the market of nanomaterials in water and wastewater treatment will include these types of NPs due their potential to EPs.

This research, as academic writing, offers an extensive overview of the current knowledge in ZnO photocatalyst alternative routes of preparation and its performance in the degradation of emerging pollutants. Green synthesis offers ecological alternatives, especially for vegetable waste that can become precursors for the synthesis of ZnO nanostructures. The article addresses a specific topic, namely, ZnO as a versatile and cost-effective photocatalyst when compared to TiO_2_. This research also provides young researchers and students with a holistic conceptualization and synthesis of the literature regarding the importance of green synthesis in ZnO photocatalysts as sustainable materials for degrading emerging pollutants.

The market research carried out by maximize market research indicates a growth of 9.1% through 2022 to 2027, reaching nearly US$ 2.7 billion, regarding Global Nanotechnology in Water Treatment (including industrial and potable water treatment) (Maximize Market Research). The most effective methods involve nanomembrane systems, essential in pollutant removal and softening, especially for pharmaceutical contaminants. In addition, nanoadsorbents are included in this report as efficient materials for a wide range of organic and inorganic pollutants. An ideal nanomaterial is the one with the smallest possible dimensions to exhibit catalytic potential and reactivity through a large specific surface area; systems that include metal oxides such as ZnO will represent the next generation of sustainable materials.

Because the risk of reaching the environment, after being used as a photocatalyst, is quite high, due to the small size, the creation of some supports that incorporate these NPs represents a viable alternative to current techniques. The most used support materials are the carbon ones, with proven properties and which in turn can be obtained from waste. Currently, the photocatalytic activity of these NPs is recognized, and these applications will represent a potential nexus on the research market.

The conventional preparation methods of ZnO NPs are associated with toxicity, which has been a restraining factor for the global ZnO NP market. However, the new trend in green synthesis is bringing attention to new perspectives regarding the acquisition and integration of these NPs. The investments in the ZnO NP sector are in expansion and linked with research and development in industry. Environmental concerns regarding EPs bring into question new industrial solutions and participants in the global market. Future researches should be developed on the cost–benefit analysis regarding the preparation methods, treatment processes, and value-added product regeneration efficiency.

### Supplementary Information

Below is the link to the electronic supplementary material.

Supplementary file1 (PDF 24 KB)

Supplementary file2 (PDF 30 KB)

Supplementary file3 (PDF 181 KB)

Supplementary file4 (PDF 29 KB)

## Data Availability

Not applicable.

## Abbreviations

AC: Activated carbon
ACE: Acetaminophen
AFM: Atomic force microscopy
ANT: Antipyrine
AOP: Advanced oxidation process
ARG: Antibiotic resistance genes
ASW: Artificial sweeteners
BPA: Bisphenol A
CEL: Cephalexin
CIP: Ciprofloxacin
CQDs: Carbon quantum dots
DBP: Disinfection byproducts
EDC: Endocrine-disrupting chemicals
ENM: Engineered nanomaterials
EPs: Emerging pollutants
FT-IR: Fourier-transform infrared spectroscopy
GO: Graphene oxide
gC_3_N_4_: Graphitic carbon nitride
HPW: Tungstophosphoric acid
IBP: Ibuprofen
ICLT: Ion exchange clinoptilolite
MB: Methylene blue
MgAC: Magnesium aminoclay
MO: Methylene orange
MP: Microplastics
MPT: Methyl parathion
NSt: Nanostars
N,S-CQDs: N,S-doped carbon quantum dots
PDM: Pendimethalin
PE: Photodegradation efficiency
PGCN: P-doped graphitic carbon nitride
PhAC: Pharmaceutically active compounds
PPCP: Personal care products
rGO: Reduced graphene oxide
ROS: Reactive oxygen species
SEM: Scanning electron microscopy
T-Ag: Triangular silver nanoplates
TEM: Transmission electron microscopy
TFL: Trifluralin
ThB: Thymol blue
UV-Vis: Ultraviolet-visible spectroscopy
XRD: X-ray diffraction
ZnO NPs: Zinc oxide nanoparticles
