# Supplementary material for: ZnO nanostructured matrix as nexus catalysts for the removal of emerging pollutants
Source: Environ Sci Pollut Res Int. 2023 Nov 3;30(54):114779–821. doi: 10.1007/s11356-023-30713-3 (PMC10682326; doi:10.1007/s11356-023-30713-3)
Supplement: Supplementary file 1 — Supplementary file1 (PDF 24 KB) [file 11356_2023_30713_MOESM1_ESM.pdf]

ELSEVIER

## Thank you for your order!

Dear Ecaterina Matei,

Thank you for placing your order through Copyright Clearance Center's RightsLink® service.

**Order Summary**

| | |
|---|---|
| Licensee: | Ecaterina Matei |
| Order Date: | Sep 27, 2023 |
| Order Number: | 5636911258478 |
| Publication: | Journal of Hazardous Materials Advances |
| Title: | Pharmaceuticals and personal care products as emerging contaminants: Need for combined treatment strategy |
| Type of Use: | reuse in a journal/magazine |
| Order Total: | 0.00 EUR |

View or print complete details of your order and the publisher's terms and conditions.

Sincerely,

Copyright Clearance Center

customercare@copyright.com
https://myaccount.copyright.com

CCC RightsLink
