# Supplementary material for: ZnO nanostructured matrix as nexus catalysts for the removal of emerging pollutants
Source: Environ Sci Pollut Res Int. 2023 Nov 3;30(54):114779–821. doi: 10.1007/s11356-023-30713-3 (PMC10682326; doi:10.1007/s11356-023-30713-3)
Supplement: Supplementary file 4 — Supplementary file4 (PDF 29 KB) [file 11356_2023_30713_MOESM4_ESM.pdf]

Thank you for your order!

Dear Ecaterina Matei,

Thank you for placing your order through Copyright Clearance Center's RightsLink® service.

Order Summary

Licensee:	Ecaterina Matei
Order Date:	May 22, 2023
Order Number:	5554180341441
Publication:	Materials Today: Proceedings
Title:	Green synthesis of ZnO nanoparticles in grape extract: Their application as anti-cancer and anti-bacterial
Type of Use:	reuse in a journal/magazine
Order Total:	0.00 EUR

View or print complete details of your order and the publisher's terms and conditions.

Sincerely,

Copyright Clearance Center

customercare@copyright.com
https://myaccount.copyright.com

RightsLink
